# Use of Nintendo Wii Balance Board for posturographic analysis of Multiple Sclerosis patients with minimal balance impairment

**DOI:** 10.1186/s12984-017-0230-5

**Published:** 2017-03-11

**Authors:** Giacomo Severini, Sofia Straudi, Claudia Pavarelli, Marco Da Roit, Carlotta Martinuzzi, Laura Di Marco Pizzongolo, Nino Basaglia

**Affiliations:** 1 School of Electrical and Electronic Engineering, University College Dublin, Dublin, Ireland; 2 Neuroscience and Rehabilitation Department, Ferrara University Hospital, Ferrara, Italy

**Keywords:** Wii Balance Board, Posturographic Analysis, Multiple Sclerosis

## Abstract

**Background:**

The Wii Balance Board (WBB) has been proposed as an inexpensive alternative to laboratory-grade Force Plates (FP) for the instrumented assessment of balance. Previous studies have reported a good validity and reliability of the WBB for estimating the path length of the Center of Pressure. Here we extend this analysis to 18 balance related features extracted from healthy subjects (HS) and individuals affected by Multiple Sclerosis (MS) with minimal balance impairment.

**Methods:**

Eighteen MS patients with minimal balance impairment (Berg Balance Scale 53.3 ± 3.1) and 18 age-matched HS were recruited in this study. All subjects underwent instrumented balance tests on the FP and WBB consisting of quiet standing with the eyes open and closed. Linear correlation analysis and Bland-Altman plots were used to assess relations between path lengths estimated using the WBB and the FP. 18 features were extracted from the instrumented balance tests. Statistical analysis was used to assess significant differences between the features estimated using the WBB and the FP and between HS and MS. The Spearman correlation coefficient was used to evaluate the validity and the Intraclass Correlation Coefficient was used to assess the reliability of WBB measures with respect to the FP. Classifiers based on Support Vector Machines trained on the FP and WBB features were used to assess the ability of both devices to discriminate between HS and MS.

**Results:**

We found a significant linear relation between the path lengths calculated from the WBB and the FP indicating an overestimation of these parameters in the WBB. We observed significant differences in the path lengths between FP and WBB in most conditions. However, significant differences were not found for the majority of the other features. We observed the same significant differences between the HS and MS populations across the two measurement systems. Validity and reliability were moderate-to-high for all the analyzed features. Both the FP and WBB trained classifier showed similar classification performance (>80%) when discriminating between HS and MS.

**Conclusions:**

Our results support the observation that the WBB, although not suitable for obtaining absolute measures, could be successfully used in comparative analysis of different populations.

**Electronic supplementary material:**

The online version of this article (doi:10.1186/s12984-017-0230-5) contains supplementary material, which is available to authorized users.

## Background

Balance maintenance is a complex task that depends on the continuous flow of proprioceptive information from the muscles, tendons, joints, skin, vestibular and visual systems toward the Central Nervous System (CNS) [1]. In individuals affected by Multiple Sclerosis (MS), the extended damage that the disease causes in the CNS leads to a decreased ability in integrating the afferent proprioceptive information, thus negatively influencing postural response and the ability to safely maintain balance [2–4]. Balance deficits are often observed in patients affected by MS and account for a great part of the disability associated with the disease [5]. In MS patients with moderate impairment, the progressive deterioration of static and dynamic balance represents a relevant symptom of the progression of the disease [6]. Previous studies have reported that 30 to 63% individuals affected by MS experience a fall event between 1 and 12 months since the onset of the disease [7]. Moreover, MS patients are at high fall risk since early after the onset of the first symptoms of the disease, even before weakness during locomotion and decreased balance become clinically relevant [8]. In addition, balance has been shown to be impaired in the MS population even before the onset of observable clinical disability [9]. Being able to detect minimal changes in the balance ability of MS patients is then of paramount importance for tracking the progression of the disease and evaluating the potential positive effects of therapy [10–12].

Balance in MS is often evaluated using clinical scales, such as Berg Balance Scale. Nevertheless these scales suffer from ceiling and/or flooring effects [13] and, in the MS population, have been shown to hold good specificity but limited sensitivity [14]. Center of Pressure (COP) related balance measures obtained from laboratory-graded force platforms (FP) represent a useful complement to the clinical scales for the evaluation of stability and risk of falling [15, 16]. Several different COP-derived features have been studied in the literature in the past years. The most common ones are sway-related measures that have been shown to change significantly in elderly [17] and impaired populations (e.g., Parkinson’s, MS, Stroke) [18–21] and to be related with fall risk [15]. More complex features have also been proposed for explaining the different open and closed loop mechanisms the CNS employs when controlling balance [22]. FP-based balance parameters can be used to better discriminate among subjects with different levels of balance impairment and/or different associated fall risk and could possibly be used to evaluate changes in balance after specific interventions [7].

Static and dynamic posturographic analyses have been proposed in the past as means to evaluate balance in MS patients. Previous studies have shown that FP-based measures show clear differences between minimally impaired MS patients and healthy controls [23–25] suggesting the possibility of using FPs to track changes in balance control before their manifestation in the clinical scales [7].

Nevertheless, the potential of employing FP-based measures as a complement for balance evaluations in MS patients is limited by the fact that commercial laboratory-grade FPs present high prices (>10 k $) and require dedicated spaces and trained personnel.

For these reasons, in recent years there has been a surge of interest into low cost applications and specifically in the use of the Nintendo Wii Balance Board (WBB) for balance assessment [26]. The WBB is equipped with four force sensors placed at the four corners of the board that are used to evaluate the distribution of the weight on the board and the vertical ground reaction forces. These values are used to estimate the position of the *x* and *y* components of the COP. The limited cost of the WBB has attracted the attention of the clinical community and its use has been investigated for both training and diagnostic means [27–36]. Few studies have compared the validity and reliability of the WBB in estimating COP path lengths in healthy individuals [26], elderly [37], individuals affected by Parkinson’s disease [38] and recently by MS [39]. All these studies showed a high reliability of the system and an acceptable comparability with respect to standard FP-based measures when measuring COP path length.

The WBB potentially allows for the development of simple and reliable balance tests that could be used to complement clinical assessments in patients at different levels of disability, including those who are likely to develop fall risk but do not show substantial impairments in the clinical balance scales. In this perspective, in this study we aim to assess the possibility of using the WBB to track changes in balance of MS patients with minimal balance impairment during static posturographic tests. Specifically, in this work we aim to: a) improve on current validation of the WBB in tracking COP related features by testing its validity and reliability with respect to a standard FP in estimating 18 standard and complex balance features; b) systematically investigate how these 18 features change in MS patients with minimal balance impairment with respect to healthy control subjects; c) test the hypothesis that the WBB could be used as reliably as a laboratory-grade FP to discriminate between HS and MS patients with minimal balance impairment during static posturographic tests. The results obtained in this study will inform on the feasibility of using the WBB as a low-cost balance assessment tool for the early detection of postural disabilities and the tracking of balance impairments in the MS population.

## Methods

### Participants

18 individuals with a MS diagnosis according to McDonald’s criteria [40, 41] (MS group, 7 males, 11 females; age = 53.6 ± 12.9 years; height = 166.1 ± 6.2 cm; body mass = 69.3 ± 13.1 kg; years after MS diagnosis = 14.3 ± 12.3) were recruited in this study. Recruitment of the patients took place at the Outpatient Clinic of the Rehabilitation Unit of Ferrara University Hospital in Ferrara, Italy. The inclusion criterion for the study was a disability rate, as calculated using the Kurtzke Expanded Disability Status Scale (EDSS) lower than 5.5, indicating the ability of the subject to stand and walk independently without assistance. Exclusion criteria for the study were the presence of neurological conditions in addition to MS, impaired cognitive functions and presence of mild dementia referred as a Mini Mental Status Examination score less than 24. Before the beginning of the instrumented balance tests, the following tests were administered to MS subjects: Time Up and Go test (TUG), Berg Balance Scale test (BBS; BBS < 45 indicates fall risk [42]) and the Unified Balance Scale test (UBS) [43]. Eighteen age-matched healthy control subjects (HS group, 6 males, 12 females; age = 52.8 ± 12.8 years; height = 167.1 ± 8.3 cm; body mass 65.4 ± 13.2 kg) were also recruited and tested in this study. Exclusion criterion for the HS group was the presence of any condition, neurologic or orthopedic, that could have an effect on their balance. All participants gave their written consent to participate in the study. All procedures were carried out according to the principles of the declaration of Helsinki. The study was approved by the Ethics Committee of the Ferrara University Hospital.

### Procedures

All testing procedures for both MS and HS were performed within a single day. A laboratory-grade FP (BERTEC Model 4080–10, BERTEC, USA) was used as the gold standard for the measurement of the balance features. Data acquired from the WBB were transferred via Bluetooth to a laptop using custom-made Labview software (National Instruments, USA). Data from the FP were sampled at 1000 Hz. The WBB has an inconsistent sampling frequency of ~100 Hz for the four force channels [44]. In our acquisition system we interpolated our data and timestamps so to achieve a constant sampling frequency of 100 Hz, as previously done in [38].

An automatic calibration procedure was employed in the WBB for resetting the non-zero unloaded values of the four load cells (similarly to [39]). All participants performed two different standing balance tasks on both the WBB and FP. The tasks consisted in standing with their eyes open and standing with their eyes closed. During both tasks subjects were asked to maintain their feet at comfortable distance apart and their hands on their hips. All subjects were instructed to remain as still as possible during all the testing sessions. These tasks are commonly used during quantitative assessment of balance and are easily executable by subjects with impaired balance. The orders of the tasks and of the testing devices were randomized across subjects.

For each combination task/device, subjects performed five consecutive trials, each lasting 60 s. Subjects were allowed to rest between trials at their convenience and were allowed an additional break before starting the set of trials with the second device. All clinical and instrumented assessments in this study were performed by the clinical staff of the Rehabilitation Unit of Ferrara University Hospital.

### Data analysis

Data from both devices were low-pass filtered at 10 Hz, using a 5^th^ order Butteworth filter to reduce unwanted noise components [45]. The *x* and *y* coordinates of the COP were calculated from the sensors of FP and WBB using formulas 1 and 2, respectively:

1 $$ \left\{\begin{array}{l} COP\_ F{P}_x=-\frac{M_y+{d}_z\cdot {F}_x}{F_z}\\ {} COP\_ F{P}_y=\frac{M_x+{d}_z\cdot {F}_y}{F_z}\end{array}\right. $$

where *M*_*x*_ and *M*_*y*_ represent the moments on the x and y axis, respectively; *F*_*x*_ and *F*_*y*_ represent the forces measured on the x and y axis, respectively; and *d*_*z*_ represents the thickness, in mm, of the tile covering the FP surface.

2 $$ \left\{\begin{array}{l} COP\_ WB{B}_x=\frac{L_x}{2}\cdot \frac{\left(\left( TR+ BR\right)-\left( TL+ BL\right)\right)}{\left( TR+ BR+ TL+ BL\right)}\\ {} COP\_ WB{B}_y=\frac{L_y}{2}\cdot \frac{\left(\left( TL+ TR\right)-\left( BL+ BR\right)\right)}{\left( TR+ BR+ TL+ BL\right)}\end{array}\right. $$

where *TL, TR, BL, BR* represent the four force sensors in the four corners of the force plate (Top, Bottom, Left and Right) and *L*_*x*_ *, L*_*y*_ represent the length of the WBB in the *x* and *y* dimensions which are 43.3 cm and 23.8 cm, respectively [44]. Equation (2) presents the formulas typically used for the calculation of the COP in the WBB native gaming applications. All COP trajectories were calculated expressed in *mm*. The 2D COP trajectories (after removal of average deviations in medio lateral (ML) and antero posterior (AP) directions) for each trial and for each subject were pooled together and plotted. The aim of this visual inspection was to assess gross differences in COP calculation across populations and measurement systems. COP trajectories were used to calculate 16 traditional balance-related features [15, 46]. All features were calculated on the middle 30 s of each trial, which is the time frame ordinarily used for the calculation of standard balance features. The features extracted included: total path length, ML and AP path length (*mm*), ML and AP range (*mm*), ML and AP root mean squared distance (*mm*), ML and AP average sway (*mm*), sway area (*mm* ^*2*^), sway speed (*mm/s*), average ML and AP speed, maximal and average radius of sway (*mm*) and area of the 95% confidence ellipse area (*mm* ^*2*^) [47]. These features are traditionally used in instrumented balance assessment and, in some cases, have been linked to fall risk in older adults and in the clinical population [46]. At risk of redundancy, we decided to calculate a substantial number of features to allow comparability between our results and those previously presented in literature. The average across the five trials of the features calculated for each trial of each condition was used to represent each subject in each experimental/measurement condition.

**Fig. 1:**
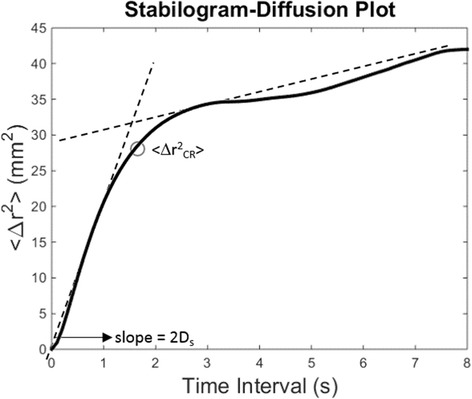
Example of the calculation of Stabilogram Diffusion Analysis features from a diffusion plot. Diffusion plots are characterized by a behavior that can be modeled as two intersecting linear functions. The parameter < Δr^2^_CR_ > is calculated as the first negative zero crossing of the derivative of the SDA line. The parameter D_s_ is calculated as half of the slope of the line best fitting the portion of the curve from 0 to < Δr^2^_CR_>

Two features extracted from the Stabilogram Diffusion Analysis (SDA) of the COP plots were also included in this study [22]. SDA analysis is generally used to derive features describing the components of the dynamic changes in the COP trajectory. In particular, SDA is used to identify components of closed and open-loop balance control at different timescales. In our analysis, we extracted two SDA parameters, namely the critical mean square displacement < Δr^2^_CR_ > that represents the threshold at which closed-loop mechanisms engage in postural control, and D_s_ that represents a measure of the stochastic activity of the COP in the short term, associated with open-loop control. The parameter < Δr^2^_CR_ > is calculated as the first negative zero crossing of the derivative of the SDA line while the parameter D_s_ as half of the slope of the line best fitting the portion of the curve from 0 to < Δr^2^_CR_ > (Fig. 1 shows an example on how the SDA parameters are calculated from the diffusion plots). SDA features were extracted, for each subject, from the average bidimensional stabilogram-diffusion plot obtained from the five trials of each condition/device. SDA features were extracted using the whole 60 s of each trial, as recommended in [48]. All analysis were performed using custom made software developed in Matlab (Mathworks, Natick, USA).

### Analysis of path lengths

A number of methodologies were used to capture different aspects and differences between the features calculated from the FP and WBB data. Most of these techniques have been used in previous works comparing FP and WBB measurements [26, 37], and have been replicated in this work to allow for comparability. Least square linear regression was used to evaluate the linear correlation between the total, ML and AP path lengths calculated from the FP and WBB. The regression was calculated from the pooled data extracted from both groups of subjects for both testing conditions. Bland-Altman plots were created for both eyes-open and eyes-closed conditions to individuate agreement between the measurements of the two devices. The plots were created by projecting the difference between the two measures (FP- WBB) against the average of the two [49]. Also in this case the data of the two groups were pooled together. Bland-Altman plots were generated for all 18 features extracted during our analysis.

### Statistical analysis

Descriptive analysis (mean ± std across trials and subjects) was used to characterize the value of the different features extracted in the analysis. A statistical analysis, performed for all 18 features in both eyes-open and eyes-closed conditions, was used to: i) assess differences between the two different groups of subjects (HS and MS) for both FP and WBB measurements; ii) compare the outcomes of the two different measurement devices for each population. This analysis was based on paired t-tests (α = 0.05) applied on the features calculated for each condition and for each subject. The p-values were adjusted for multiple comparisons (two) using Bonferroni’s correction. The Spearman correlation coefficient (ρ) was used to analyze the correlation between the features extracted using the FP and WBB. Significant (α = 0.05) values of correlation with *ρ* > 0.5 were considered as representative of a high degree of correlation [37]. The reliability across the two measurement devices was assessed using a two-way random-effects single measure (as average of the five trials) Intraclass Correlation Coefficients (ICC) model. For ICC, values > 0.75 were used to indicate excellent reliability, while values 0.4–0.74 were used to indicate moderate-to-high reliability [26, 50].

### Classification

We evaluated how the features extracted from the FP and WBB devices can be used to discriminate between HS and MS subjects during both the eyes-open and eyes-closed conditions. In this analysis we trained four classifiers (open/closed and FP/WBB) and assessed the outcome of classification on our dataset. The classifiers were based on a Support Vector Machine (SVM) design with Gaussian Kernel. We chose to use the SVM design given the success of this approach on binomial classification problems. The choice of kernel and parameters were made so to maximize the classification performance for the FP dataset (data not presented). After an initial screening of the SVM classification performance we decided to train the classifiers using only the classic features, thus excluding the SDA-based features. Features were standardized before being fed to the classifier. Classifiers were 10-fold trained/validated for 100 iterations. The average classification ratio for the 100 classifier trained in each condition was calculated.

## Results

### Participants

The MS group presented the following results for the clinical tests: EDSS = 3.4 ± 2.1; TUG = 8.4 ± 2.6 s; BBS = 53.3 ± 3.1; UBS = 80.2 ± 8.1. The subjects were able to walk independently and presented minimal fall risk as highlighted by the results of the BBS exam [42].

### Path lengths

**Fig. 2:**
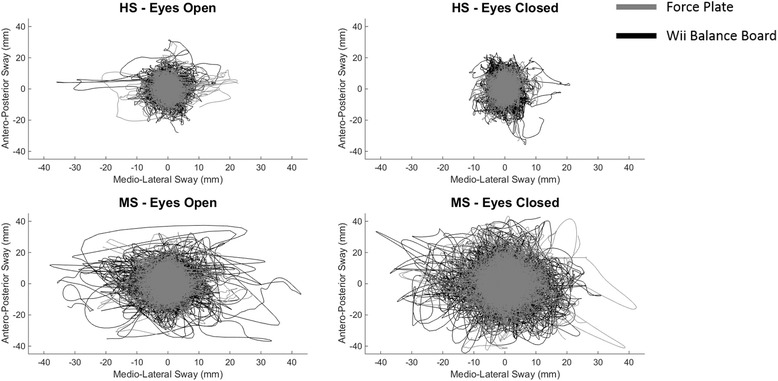
Comparison of the stabilograms for each combination of task/population. Each plot presents the superimposed detrended stabilograms for each task of each subject. *Grey lines* represent COP trajectories obtained from the FP, while *black lines* represent those obtained from the WBB. Stabilograms obtained from the WBB are generally wider than those obtained from FP. Moreover, while for both the WBB and the FP in HS there are limited differences between the stabilograms relative to eyes-open and eyes-closed conditions, for MS stabilograms relative to the eyes-closed condition are generally more extended than those relative to eyes-open condition

Figure 2 shows the pooled stabilograms obtained from the FP and WBB for the different groups in the different conditions. In general, the stabilograms obtained from the WBB (black) were shown to be wider than the ones obtained from the FP (grey). We did not observe obvious differences between the eyes-open and eyes-closed conditions in the HS. In the MS sample the stabilograms for the eyes-closed condition were wider than those for the eyes-open condition, as expected. This observation was confirmed for both the FP and the WBB.

**Fig. 3:**
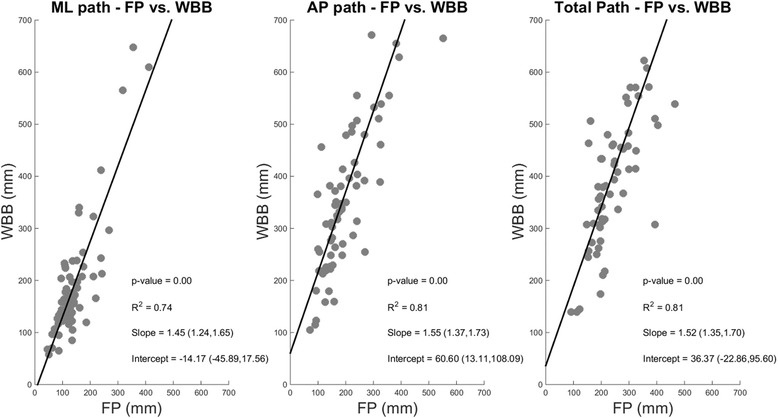
Linear Correlation analysis between path lengths (ML, AP and Total) calculated using the FP and WBB. Data for HS and MS and for both testing conditions (eyes-open and eyes-closed) have been pooled together in this analysis. Parameters of the fitting (p-value, R^2^, Slope and Intercept of the fitting) are presented in the plot for each analysis. WBB and FP present similar linear trends for both ML and AP COP directions, consistent with an overestimation of the directional sway in the WBB with respect to FP

**Fig. 4:**
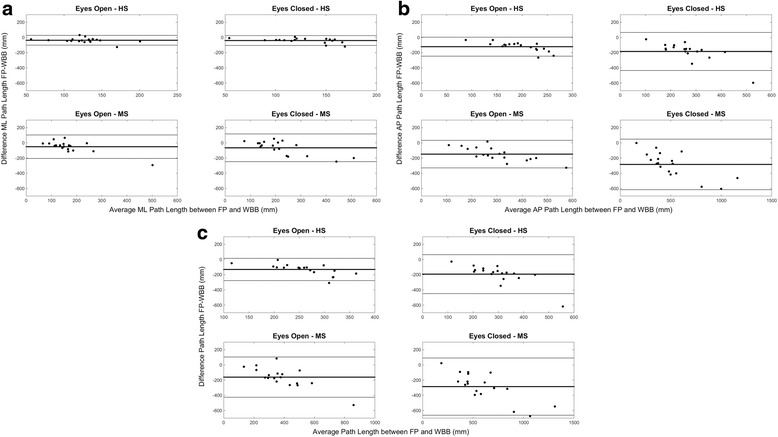
Bland-Altman Plots for each combination of task/population. Subplot (**a**) presents the results for ML direction, subplot (**b**) for the AP direction and subplot (**c**) for the total path length. Y axis of each plot presents the difference between FP and WBB, while X axis presents the average between the two measures. All the plots show a decrease in accuracy of the WBB with respect to the FP as the magnitude of the estimated value increases

The analysis of the linear correlation for the total, ML and AP path length measures obtained from the FP and the WBB (Fig. 3) confirmed the preliminary observation made in the stabilogram plots. In fact, for all three measures, we observed a slope > 1 (1.52 for total path, 1.45 for ML and 1.55 for AP) for the linear regression functions, indicating a consistent over-estimation of unidimensional and bidimensional path lengths from the WBB with respect to the FP. The same result was observed also in the Bland-Altman plots (Figs. 4a, b and c). For the path length in the ML direction (Fig. 4a) we observed a consistent average negative bias of about 50 to 80 mm across groups and conditions indicating over-estimation of the parameter by the WBB. We also observed a linear negative trend that is more pronounced in the MS group, possibly due to the fact that this population showed a higher path length measure in both conditions. For the path length in the AP direction (Fig. 4b), which during standing balance is generally higher than the one in ML direction in both HS and MS, we observed a negative bias (~110 to 300 mm) that is proportional to the variability observed. Also in this case a clear negative linear trend is observable in all four plots. The same results are observed in the total path length (Fig. 4c). In the Additional file 1 we present the Bland-Altman plots for all the 18 features extracted.

### Differences among devices and populations

**Fig. 5:**
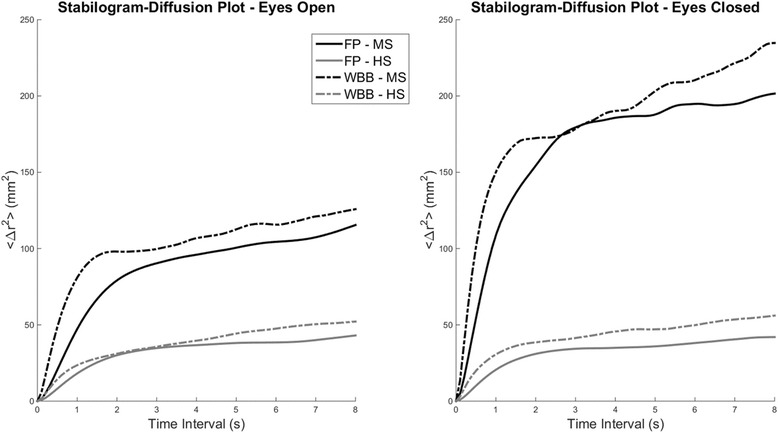
Stabilogram Diffusion Plots for eyes-open and eyes-closed conditions. *Black lines* represent data from MS, while *grey lines* represent data from HS. *Bold lines* represent data obtained using the FP, while *dash-dot lines* present data obtained using the WBB. *Each line* represents the average Stabilogram Diffusion Plot across all the subjects for each measurement system in each condition. MS patients present higher diffusion plot curves that are consistent with increased sway. Diffusion plots obtained from WBB follow similar trends with respect to those obtained from FP

Table 1 presents the across-subjects average values for each single feature in each condition, together with the statistical comparisons of the means between different groups and measurement devices. For the eyes-open condition, as expected, we observed higher values in all features for the MS sample with respect to the HS. These differences were statistically significant for all features with the exclusion of the ML path length, speed and sway area. Although the WBB overestimates most of the features, we found significant differences between the FP and WBB data only for the path lengths (total and AP for MS and total, ML and AP for HS). For the eyes-closed condition we observed statistically significant differences between HS and MS for all features for both FP and WBB-based measures. For the between-devices analysis we found significant differences for the same features as the eyes-open condition, with the addition of the ML range and the maximal radial position for HS. The analysis of the Spearman coefficient (Table 2) indicates that the features are highly correlated (ρ > 0.5) between the two measurement devices for all parameters with the exclusion of the ML path length and speed, AP range and all the SDA parameters for the eyes-open condition (both HS and MS). For the eyes-closed condition all features have been shown to be highly correlated in both MS and HS. Concurrent validity, as estimated using the ICC analysis (Table 3), was moderate-to-high for almost all features in most conditions, with the exclusion of the SDA ones. ICC was found to increase with the variability of the task/condition, holding better results in the task/group with more variability (MS, eyes-closed). SDA plots (Fig. 5) show distinct trends for HS and MS that are consistent between FP and WBB for both eyes-open and eyes-closed conditions.

**Table 1:** Mean ± standard deviation values for each features for each group (HS and MS) and measurement system (FP and WBB). The top table shows the results for the eyes-open condition, the bottom table for the eyes-closed condition. Results of statistical analysis (*t*-test, α = 0.05) is presented. Column *FP - HS vs MS (p)* presents the results of the statistical analysis between HS and MS populations for the data extracted using the FP. Column *WBB - HS vs MS (p)* presents the results of the statistical analysis between HS and MS populations for the data extracted using the WBB. Column *HS - FP vs WBB (p)* presents the results of the statistical analysis between FP and WBB for the HS population. Column *MS- FP vs WBB (p)* presents the results of the statistical analysis between FP and WBB for the MS population. For each column the p values are presented. * denotes *p*-value < 0.05; ** denotes a *p*-value < 0.01

	FP – HS	FP - MS	WBB - HS	WBB - MS	FP - HS vs MS (p)	WBB - HS vs MS (p)	HS - FP vs WBB (p)	MS - FP vs WBB (p)
Eyes-open								
Total Path (mm)	202.5 ± 46.3	302.5 ± 113.8	332.1 ± 86.2	477.6 ± 206.0	0.003(**)	0.018(*)	<0.001(**)	0.007(**)
ML Path (mm)	113.2 ± 29.5	148.8 ± 69.1	152.1 ± 41.9	206.5 ± 132.3	0.102	0.199	0.006(**)	0.208
AP Path (mm)	142.4 ± 36.3	230.5 ± 84.0	261.2 ± 73.0	381.3 ± 146.3	<0.001(**)	0.007(**)	<0.001(**)	0.001(**)
ML Range (mm)	9.5 ± 3.9	17.4 ± 11.2	11.0 ± 4.8	20.7 ± 13.0	0.015(*)	0.010(*)	0.532	0.673
AP Range (mm)	17.8 ± 5.8	30.6 ± 11.8	21.3 ± 6.0	31.0 ± 11.8	<0.001(**)	0.008(**)	0.159	0.993
ML RMS (mm)	1.8 ± 0.7	3.4 ± 1.8	2.0 ± 0.8	3.8 ± 2.2	0.003(**)	0.007(**)	0.696	0.798
AP RMS (mm)	3.7 ± 1.2	6.4 ± 2.3	4.2 ± 1.3	6.0 ± 2.3	<0.001(**)	0.017(*)	0.428	0.842
ML Average Disp (mm)	1.4 ± 0.6	2.7 ± 1.3	1.6 ± 0.7	3.0 ± 1.7	0.002(**)	0.006(**)	0.674	0.776
AP Average Disp (mm)	3.0 ± 0.9	5.1 ± 1.8	3.4 ± 1.1	4.7 ± 1.9	<0.001(**)	0.025(*)	0.404	0.823
Swept Area (mm^2^)	149.4 ± 95.5	530.7 ± 711.6	201.8 ± 138.0	598.6 ± 621.5	0.061	0.024(*)	0.35	0.943
Sway Speed (mm/s)	10.2 ± 2.3	15.2 ± 5.7	11.1 ± 2.9	16.3 ± 6.8	0.003(**)	0.010(*)	0.518	0.846
ML Average Sp (mm/s)	5.7 ± 1.5	7.5 ± 3.5	5.1 ± 1.4	7.1 ± 4.4	0.102	0.148	0.37	0.942
AP Average Sp (mm/s)	7.1 ± 1.8	11.6 ± 4.2	8.7 ± 2.4	13.0 ± 4.8	<0.001(**)	0.004(**)	0.073	0.59
Max Radial Pos (mm)	11.0 ± 3.7	19.0 ± 8.0	12.6 ± 4.0	18.9 ± 8.1	<0.001(**)	0.011(*)	0.373	1
Average Radial Pos (mm/s)	3.6 ± 1.1	6.3 ± 2.3	4.1 ± 1.3	6.2 ± 2.6	<0.001(**)	0.008(**)	0.46	0.993
Area Ellipse (mm^2^)	89.2 ± 56.0	286.2 ± 260.3	110.3 ± 73.1	315.5 ± 299.3	0.007(**)	0.016(*)	0.561	0.94
D_s_ (mm^2^/s)	4.7 ± 3.6	13.2 ± 10.4	6.7 ± 5.2	26.4 ± 35.5	0.005(**)	0.052	0.346	0.262
<Δr^2^_CR_ > (mm^2^)	36.4 ± 26.7	86.5 ± 78.1	37.2 ± 25.9	103.4 ± 80.8	0.029(*)	0.004(**)	0.994	0.776
Eyes-closed								
Total Path (mm)	231.9 ± 63.7	461.2 ± 208.4	414.7 ± 148.2	750.2 ± 359.4	<0.001(**)	0.002(**)	<0.001(**)	0.011(*)
ML Path (mm)	109.1 ± 27.1	197.8 ± 79.7	148.3 ± 40.1	260.9 ± 138.6	<0.001(**)	0.004(**)	0.003(**)	0.196
AP Path (mm)	180.3 ± 58.4	372.4 ± 192.5	356.2 ± 143.1	648.9 ± 327.4	<0.001(**)	0.003(**)	<0.001(**)	0.008(**)
ML Range (mm)	8.1 ± 3.3	23.2 ± 15.4	9.7 ± 4.4	24.6 ± 14.5	<0.001(**)	<0.001(**)	0.404	0.956
AP Range (mm)	18.7 ± 6.2	38.0 ± 14.0	25.6 ± 8.0	43.2 ± 16.2	<0.001(**)	<0.001(**)	0.014(*)	0.528
ML RMS (mm)	1.6 ± 0.7	4.5 ± 2.8	1.8 ± 0.8	4.7 ± 2.7	<0.001(**)	<0.001(**)	0.609	0.975
AP RMS (mm)	4.0 ± 1.3	7.9 ± 3.1	4.9 ± 1.6	8.1 ± 3.2	<0.001(**)	0.001(**)	0.14	0.981
ML Average Disp (mm)	1.3 ± 0.5	3.5 ± 2.1	1.5 ± 0.6	3.7 ± 2.2	<0.001(**)	<0.001(**)	0.581	0.956
AP Average Disp (mm)	3.3 ± 1.1	6.4 ± 2.5	3.9 ± 1.3	6.5 ± 2.6	<0.001(**)	0.002(**)	0.187	0.993
Swept Area (mm^2^)	129.0 ± 92.2	775.7 ± 787.5	209.6 ± 150.9	937.5 ± 797.9	0.003(**)	0.001(**)	0.119	0.792
Sway Speed (mm/s)	11.6 ± 3.2	23.2 ± 10.5	13.9 ± 4.9	25.8 ± 12.1	<0.001(**)	<0.001(**)	0.222	0.731
ML Average Sp (mm/s)	5.5 ± 1.4	9.9 ± 4.0	5.0 ± 1.3	9.0 ± 4.9	<0.001(**)	0.003(**)	0.444	0.803
AP Average Sp (mm/s)	9.0 ± 2.9	18.7 ± 9.7	11.9 ± 4.7	22.3 ± 10.9	<0.001(**)	0.001(**)	0.073	0.512
Max Radial Pos (mm)	10.7 ± 3.5	23.7 ± 10.1	14.5 ± 4.5	26.0 ± 10.4	<0.001(**)	<0.001(**)	0.017(*)	0.745
Average Radial Pos (mm/s)	3.7 ± 1.3	8.0 ± 3.4	4.5 ± 1.4	8.2 ± 3.4	<0.001(**)	<0.001(**)	0.231	0.981
Area Ellipse (mm^2^)	82.5 ± 61.8	478.0 ± 450.6	109.4 ± 80.2	515.5 ± 437.7	0.002(**)	<0.001(**)	0.462	0.961
D_s_ (mm^2^/s)	6.1 ± 5.6	32.9 ± 32.1	9.2 ± 9.0	48.4 ± 42.4	0.003(**)	0.001(**)	0.399	0.401
<Δr^2^_CR_ > (mm^2^)	34.7 ± 25.3	186.8 ± 163.6	41.0 ± 29.0	184.6 ± 141.9	<0.001(**)	<0.001(**)	0.744	0.999

**Table 2:** Spearman Coefficient Analysis for each feature in each combination of condition and population. Only values with significance level *p* < 0.05 are presented (ns indicates a combination of feature/condition for which Spearman coefficient was not significant). Values for *ρ* > 0.5 indicate high validity and are presented in bold

	Eyes-open - HS	Eyes-open - MS	Eyes-closed - HS	Eyes-closed – MS
Total Path	**0.78**	**0.51**	**0.81**	**0.7**
ML Path	ns	0.43	**0.73**	**0.57**
AP Path	**0.79**	**0.59**	**0.78**	**0.73**
ML Range	**0.72**	**0.75**	**0.59**	**0.64**
AP Range	ns	0.42	**0.9**	**0.78**
ML RMS	**0.72**	**0.75**	**0.51**	**0.55**
AP RMS	**0.79**	**0.63**	**0.87**	**0.75**
ML Average Disp	**0.68**	**0.7**	**0.5**	**0.54**
AP Average Disp	**0.79**	**0.59**	**0.84**	**0.78**
Swept Area	**0.73**	**0.59**	**0.8**	**0.79**
Sway Speed	**0.78**	**0.51**	**0.7**	**0.7**
ML Average Sp	ns	0.43	**0.7**	**0.59**
AP Average Sp	**0.81**	**0.59**	**0.7**	**0.73**
Max Radial Pos	**0.82**	**0.6**	**0.93**	**0.76**
Average Radial Pos	**0.83**	**0.72**	**0.87**	**0.82**
Area Ellipse	**0.7**	**0.62**	**0.61**	**0.74**
D_s_	ns	0.28	**0.71**	**0.71**
<r^2^_CR_>	0.42	ns	**0.77**	**0.89**

**Table 3:** Intraclass Coefficient Analysis for each feature for each combination of condition and population. Values of ICC > 0.4 (italic) indicate moderate reliability. Values of ICC > 0.74 (bold + italic) indicate high reliability

	Eyes-open - HS	Eyes-open - MS	Eyes-closed - HS	Eyes-closed – MS
Total Path	*0.44 (−0.02, 0.75)*	*0.72 (0.39, 0.88)*	*0.43 (−0.03, 0.74)*	***0.79 (0.53, 0.92)***
ML Path	*0.58 (0.18, 0.82)*	*0.72 (0.39, 0.88)*	*0.56 (0.15, 0.81)*	*0.70 (0.37, 0.88)*
AP Path	0.38 (−0.10, 0.71)	*0.73 (0.41, 0.89)*	*0.41 (−0.06, 0.73)*	***0.81 (0.55, 0.92)***
ML Range	*0.72 (0.39, 0.88)*	***0.84 (0.63, 0.94)***	*0.64 (0.26, 0.85)*	***0.76 (0.47, 0.90)***
AP Range	*0.43 (−0.03, 0.74)*	***0.91 (0.77, 0.96)***	*0.71 (0.38, 0.88)*	***0.91 (0.77, 0.96)***
ML RMS	*0.71 (0.37, 0.88)*	***0.76 (0.46, 0.90)***	*0.53 (0.10, 0.79)*	*0.73 (0.41, 0.89)*
AP RMS	*0.62 (0.23, 0.84)*	***0.88 (0.71, 0.95)***	***0.79 (0.52, 0.92)***	***0.92 (0.79, 0.97)***
ML Average Disp	*0.67 (0.30, 0.86)*	*0.72 (0.39, 0.89)*	*0.43 (−0.03, 0.74)*	*0.66 (0.28, 0.86)*
AP Average Disp	*0.63 (0.24, 0.84)*	***0.89 (0.73, 0.96)***	***0.82 (0.58, 0.93)***	***0.91 (0.78, 0.97)***
Swept Area	*0.53 (0.10, 0.79)*	***0.79 (0.52, 0.92)***	*0.70 (0.35, 0.88)*	***0.76 (0.47, 0.90)***
Sway Speed	*0.54 (0.11, 0.80)*	***0.84 (0.62, 0.94)***	*0.55 (0.12, 0.80)*	***0.84 (0.62, 0.94)***
ML Average Sp	*0.61 (0.21, 0.83)*	***0.84 (0.63, 0.94)***	*0.63 (0.24, 0.84)*	*0.73 (0.42, 0.89)*
AP Average Sp	*0.48 (0.03, 0.77)*	***0.85 (0.65, 0.94)***	*0.53 (0.09, 0.79)*	***0.86 (0.67, 0.95)***
Max Radial Pos	*0.48 (0.03, 0.77)*	***0.87 (0.68, 0.95)***	*0.71 (0.38, 0.88)*	***0.86 (0.66, 0.94)***
Average Radial Pos	*0.73 (0.41, 0.89)*	***0.89 (0.72, 0.96)***	***0.81 (0.57, 0.93)***	***0.89 (0.74, 0.96)***
Area Ellipse	*0.51 (0.07, 0.78)*	***0.86 (0.66, 0.94)***	*0.68 (0.32, 0.87)*	*0.70 (0.36, 0.88)*
D_s_	0.11 (−0.36, 0.54)	*0.40 (−0.06, 0.73)*	0.21 (−0.27, 0.61)	*0.56 (0.14, 0.81)*
<Δr^2^_CR_>	0.34 (−0.14, 0.69)	0.39 (−0.08, 0.72)	***0.90 (0.76, 0.96)***	***0.74 (0.42, 0.89)***

### SVM classification

**Fig. 6:**
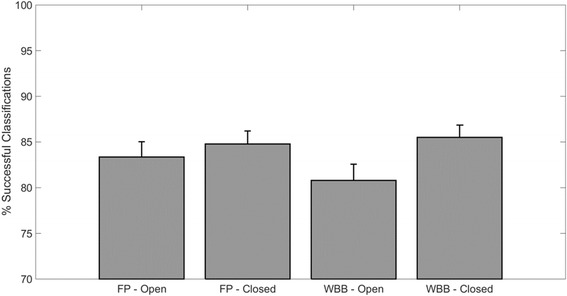
Classification performance for the 4 different set of SVM-based classifiers trained. Classifiers have been trained for each combination of task/measurement device. Y axis shows the percentage of successful classifications for each set of classifiers. All classifiers show similar accuracy. Classifier built from the eyes-closed database for both WBB and FP yield classification performance >80%

The classification rates of the different classifiers trained on the different tasks/measurement systems are shown in Fig. 6. As expected, the classifiers trained on the eyes-closed condition perform better than the ones trained in the eyes-open condition, possibly due to the higher variability observed in that dataset. FP and WBB classifiers trained on closed eyes features show similar classification rates (85 ± 1.7 for FP, 85.5 ± 1.4 for WBB) while for the eyes-open dataset the FP trained classifier yields slightly better results (83.3 ± 1.7) with respect to the WBB trained one (80.6 ± 1.8).

## Discussion

In this study we have tested the validity and reliability of the WBB when estimating several different COP-derived features, commonly used in posturographic analysis, with respect of a laboratory-grade FP. We also investigated how these features change between HS and MS with minimal balance impairment and we finally tested the hypothesis that the WBB could be reliably used to discriminate between HS and MS during static posturographic tests.

### Path length analysis and validity and reliability of estimation of posturographic features

The results presented in this study confirm and extend what has been shown in the past few years in literature investigating the validity and reliability of the WBB in young [26], elderly [37] and impaired population [38, 39]. Most of the previous studies aiming at assessing the use of the WBB for instrumented posturography have used, as a main outcome measure, the total COP path length. All the studies demonstrated good-to-excellent validity and reliability when using the WBB in different populations, including MS [39]. Our results further confirm this observation. We showed that there is a linear relation between the total, ML and AP path length as measured using the FP or the WBB. This linear relation is characterized by a slope of ~1.5 that is consistent across the different tasks and the two observed populations. The Bland-Altman plots on the path lengths (ML, AP and total) further reinforced this observation, as highlighted by the presence of considerable biases between the FP and WBB measures that suggest a variability-dependent overestimation of the path lengths in the WBB.

Although the overall measure for these three parameters is significantly different in most of the conditions we tested (with the exclusion of ML path length in testing conditions characterized by higher variability), this does not translate in significant differences between FP and WBB measurement in most of the derivate measures. We did not observe, in fact, significant differences in most of the features for all conditions. Interestingly we did not notice significant differences in any of the features other than the total path and AP path length for the MS population, in both the eyes-open and eyes-closed conditions.

Contrarily with what observed by Leach and colleagues [51] in their instrumented validation study, we observed a higher discrepancy in measurements for the AP rather than the ML direction, but this is possibly related to the bigger biases and variability observed in the AP direction during human balance tasks. Spearman and ICC coefficient analysis confirmed high validity and moderate to high reliability for most features with the exclusion of the SDA ones (that show ICC values > 0.4 only for the MS in eyes-closed condition). The estimation of SDA features, however, is usually performed on diffusion plots obtained from more trials with respect to the number we have used in our analysis [22, 48]. This discrepancy may explain the differences we observed. Nevertheless, we were able to observe consistent trends between MS and HS for both FP and WBB measurements, thus suggesting the possibility of performing SDA analysis also with the WBB.

With respect to the study by Castelli et al. [39], that tested the use of the WBB for estimating path length in a vast sample of MS patients, we were able to extend the validation of the WBB for several other standard and complex features, while comparing the WBB with a laboratory grade FP. Moreover, although the sample of patients analyzed in that work is characterized by a similar level of clinical impairment as defined by the EDSS, the sample we analyzed was specifically characterized by limited balance impairment (as expressed through standard clinical scales), while no information on this regard is presented in Castelli et al. The results we obtained are in full accordance with those observed in [39] and complement on our current knowledge on the use of the WBB for the estimation of balance in MS.

Specifically our results seem to confirm the observation that the WBB can be effectively used in situations where accuracy is not of paramount importance. In our study, in fact, the WBB performs more consistently with the FP for the MS group and in the eyes-closed condition, where the overall variability of the datasets is higher, while performance is lower in the HS group in the eyes-open condition, where, although the stabilogram plots are comparable (see Fig. 2) measurements and related features are less consistent.

### Comparison between healthy subjects and multiple sclerosis patients

In our study we compared the FP and WBB measures of a sample of MS patients with limited balance impairments (BBS = 53.3 ± 3.1 on a maximum of 56) and a group of age-matched HS. Under the definition of the BBS, our sample is expected to represent a population of patients presenting low risk of falling (BBS > 45). BBS, on the other hand, is known to present a ceiling effect [13, 43], that could possibly explain the differences observed in instrumented analysis between the HS and our sample of high-BBS patients. In our analysis we observed significant differences between the balance features calculated from the HS and the MS for all the traditional features (with the exclusion of ML path length and average speed in the eyes-open condition). The same differences were observed for both the FP and WBB. For the SDA features, to the authors’ knowledge, this analysis has never been extensively applied in the MS population. Our analysis unravels distinct patterns between HS and MS. In particular we notice a significant increase in both < Δr^2^_CR_ > and D_s_. An increase in < Δr^2^_CR_ > indicates a tendency to switch from open-loop to closed-loop control at bigger excursions, while an increase in *Ds* denotes a tendency of the COP trajectory towards randomly walking around a fixed equilibrium point [22, 52]. These two parameters had been shown to increase in MS with respect to healthy subjects in a previous study [53] and had been related to impaired postural control. In our work, the trends observed in the SDA are consistent between FP and WBB, and no statistical difference has been noted for these parameters between the two measurement devices. These observations suggest the validity of using the WBB for relative measurements between two different populations using the same device, as suggested by Bartlett and colleagues in [44].

### Classification of healthy subjects and multiple sclerosis patients

In this work we analyzed how well the features extracted from both the FP and WBB can be used to train a classifier whose aim is to distinguish between the HS and MS groups. This analysis is suggestive in the scenario of using objective measures from instrumented balance analysis for the development of complementary diagnostic tools for balance impairments. Although the classification rate per-se is relatively acceptable and could not, in this specific case, be successfully used clinically, it is interesting to notice that the classifiers trained on FP and WBB data yield the same classification performance. This result further confirms the usefulness of the WBB in relative measures. Moreover our results encourage on the possible use of classifiers to distinguish between patient populations with pre-defined different levels of impairment. In such scenario the WBB could represent a valuable and informative tool in the clinical environment. The classification analysis herein presented is preliminary and we acknowledge the necessity to develop specific protocols for more informative studies on this line of study. However, the preliminary results presented are informative on the possibility of implementing such classifiers on a WBB rather than on a more expensive FP.

### Limitations of this study

It needs to be acknowledged that, since we did not acquire data with the FP and WBB simultaneously, the data and features that we analyzed are not directly comparable and part of the variability observed between the devices could be due to the standard variability that is observed when evaluating multiple balance sessions. On the other hand, the consistent trends (between groups and conditions) that we observed in our results seem to confirm the validity of our analysis and the usability of the WBB in relative measurments. To improve the comfort of the patients during the FP and WBB tests we did not standardize the foot position for each subject through the tests. Instead we asked the subjects to keep the feet at a comfortable distance. This design choice could introduce biases, especially in the estimation of ML features of the COP. For the SVM analysis it is necessary to point out that our classification analysis has intrinsic technical and conceptual limitations that limit its general validity. Such classifier would be in fact of limited use in the clinical environment and our limited dataset (2 groups, 18 subjects per group) do not represent a valid representative sample. Moreover, we limited our analysis to standard static posturographic tests. In the scenario of the development of a classifier for discriminating between HS and MS patients it would be useful to add additional dynamic posturography and cognitive tests.

## Conclusions

In this study we investigated the possibility of using the WBB to track changes in balance of minimally impaired MS patients during static posturographic tests. To achieve this aim we validated the use of WBB for the estimation of 18 different balance-related features, thus extending the analysis with respect to previous works in literature. We have shown that balance features extracted from static posturographic analysis vary significantly between HS and MS with limited balance impairment. Moreover, the features estimated from WBB measurements follow the same trends and in most case do not present significant differences with respect to the one calculated using by traditional laboratory-grade FP. Finally we have also shown that WBB features can be used to train classifiers that yield the same performance as analogous ones trained with features extracted from a FP, suggesting the possibility of using the WBB for the development of simple and cost-effective complementary balance tests. We conclude that the WBB is not suitable for obtaining absolute measures of balance-related parameters, but can be successfully used as a cheap mean to obtain a vast range of measures between different populations and may represent a valid alternative to more expensive technologies in the clinical setting.

## Additional file

Additional file 1:
**S1-15.** Bland-Altman Plots for all the features extracted from both the FP and the WBB. Y axis of each plot presents the difference between FP and WBB, while X axis presents the average between the two measures. The plots show a consistent trend of overestimation of the features extracted from the WBB data characterized by a negative bias (bold lane). Most features also present a linear trend whereas the difference between WBB and FP measurements increases with the magnitude of the feature. (ZIP 1255 kb)

## Abbreviations

AP: Antero posterior
BBS: Berg balance scale
COP: Center of pressure
EDSS: Expanded disability status scale
FP: Force plate
HS: Healthy subjects
ICC: Intraclass correlation coefficient
ML: Medio lateral
MS: Multiple sclerosis
SDA: Stabilogram diffusion analysis
SVM: Support vector machines
UBS: Unified balance scale
WBB: Wii balance board
