# Supplementary figures and images for: Use of Nintendo Wii Balance Board for posturographic analysis of Multiple Sclerosis patients with minimal balance impairment
Source: J Neuroeng Rehabil. 2017 Mar 11;14:19. doi: 10.1186/s12984-017-0230-5 (PMC5346266; doi:10.1186/s12984-017-0230-5)

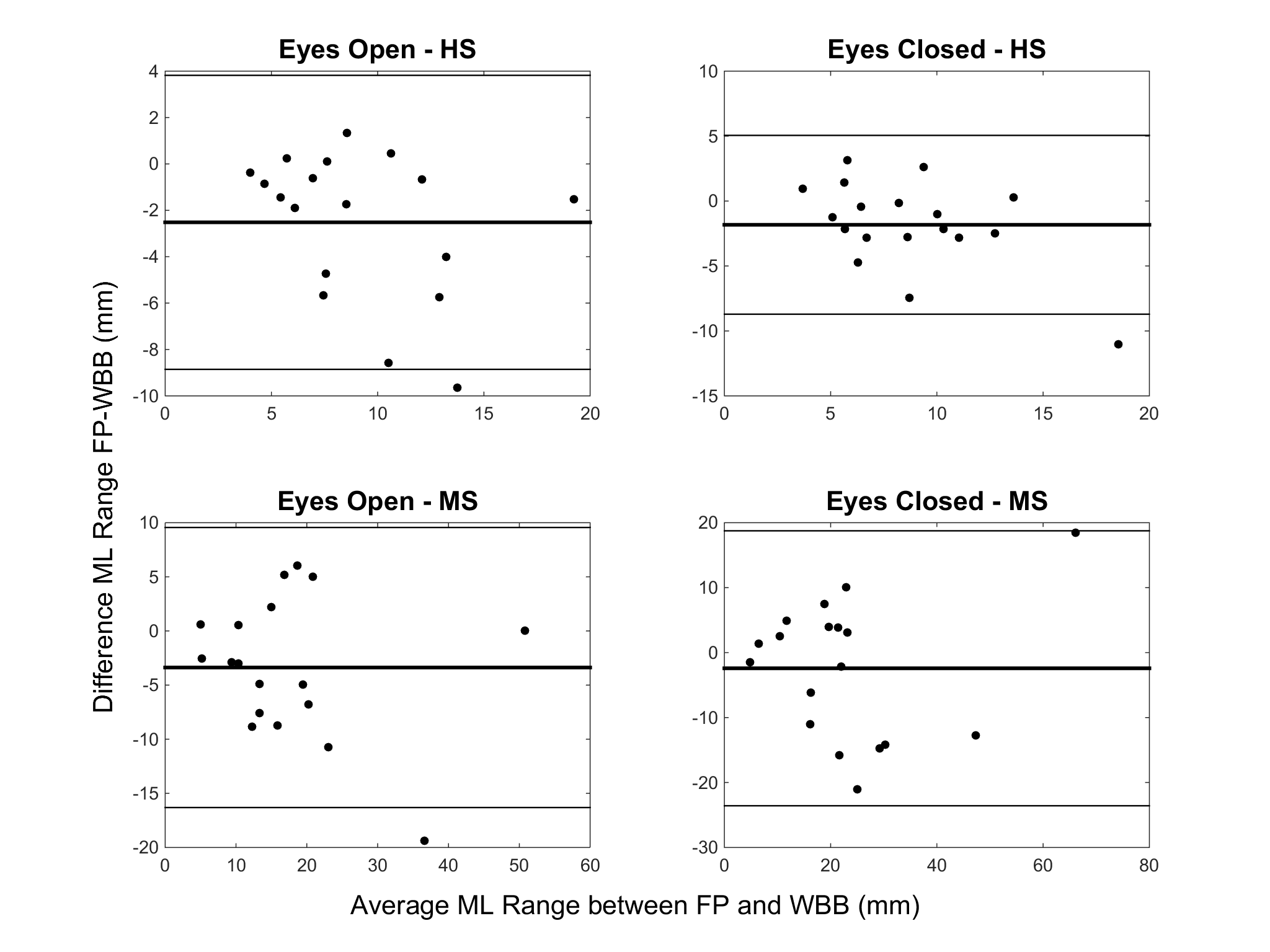

Supplement: Additional file 1: — S1-15. Bland-Altman Plots for all the features extracted from both the FP and the WBB. Y axis of each plot presents the difference between FP and WBB, while X axis presents the average between the two measures. The plots show a consistent trend of overestimation of the features extracted from the WBB data characterized by a negative bias (bold lane). Most features also present a linear trend whereas the difference between WBB and FP measurements increases with the magnitude of the feature. (ZIP 1255 kb) [file 12984_2017_230_MOESM1_ESM.zip › S1.png]

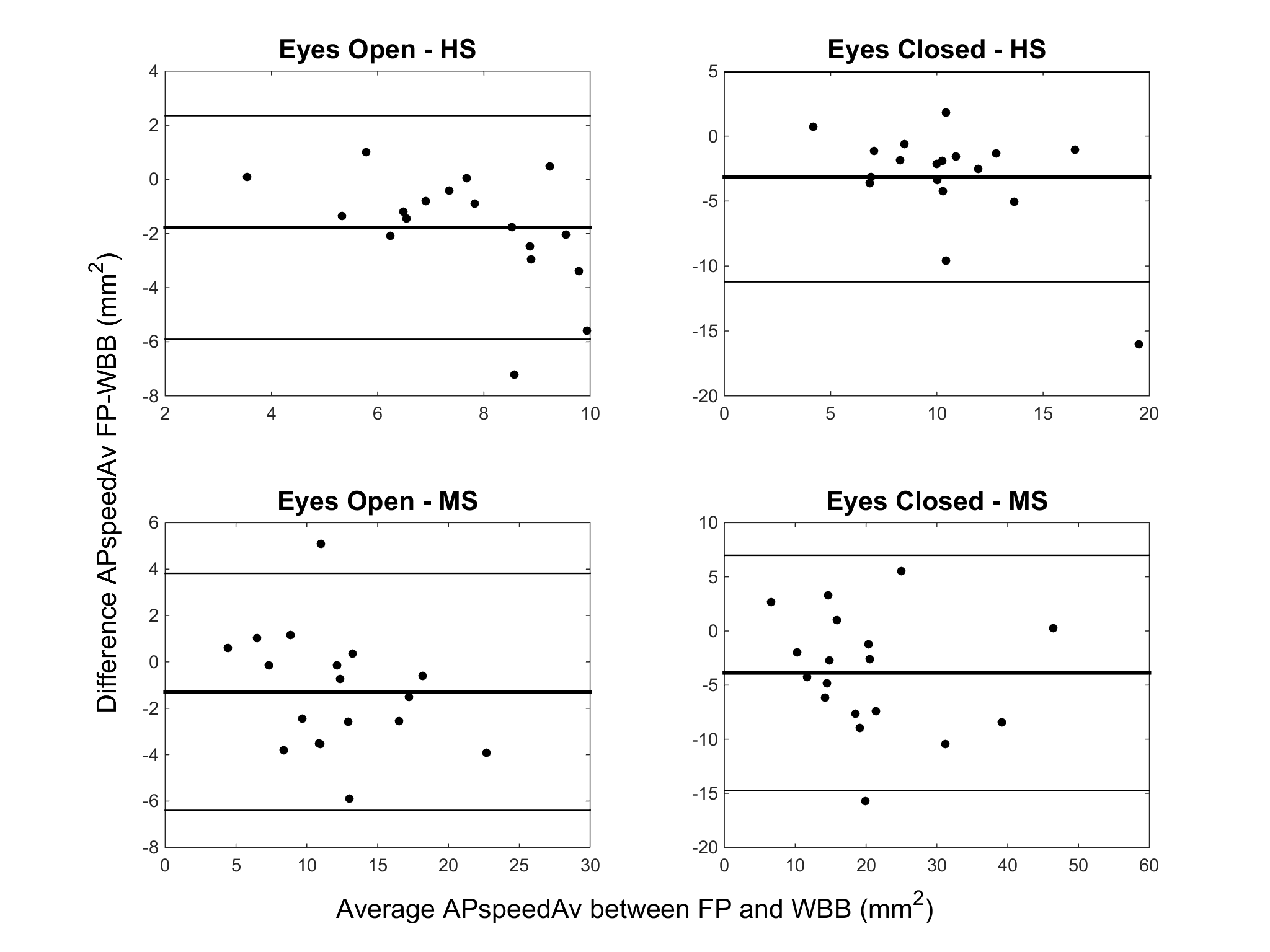

Supplement: Additional file 1: — S1-15. Bland-Altman Plots for all the features extracted from both the FP and the WBB. Y axis of each plot presents the difference between FP and WBB, while X axis presents the average between the two measures. The plots show a consistent trend of overestimation of the features extracted from the WBB data characterized by a negative bias (bold lane). Most features also present a linear trend whereas the difference between WBB and FP measurements increases with the magnitude of the feature. (ZIP 1255 kb) [file 12984_2017_230_MOESM1_ESM.zip › S10.png]

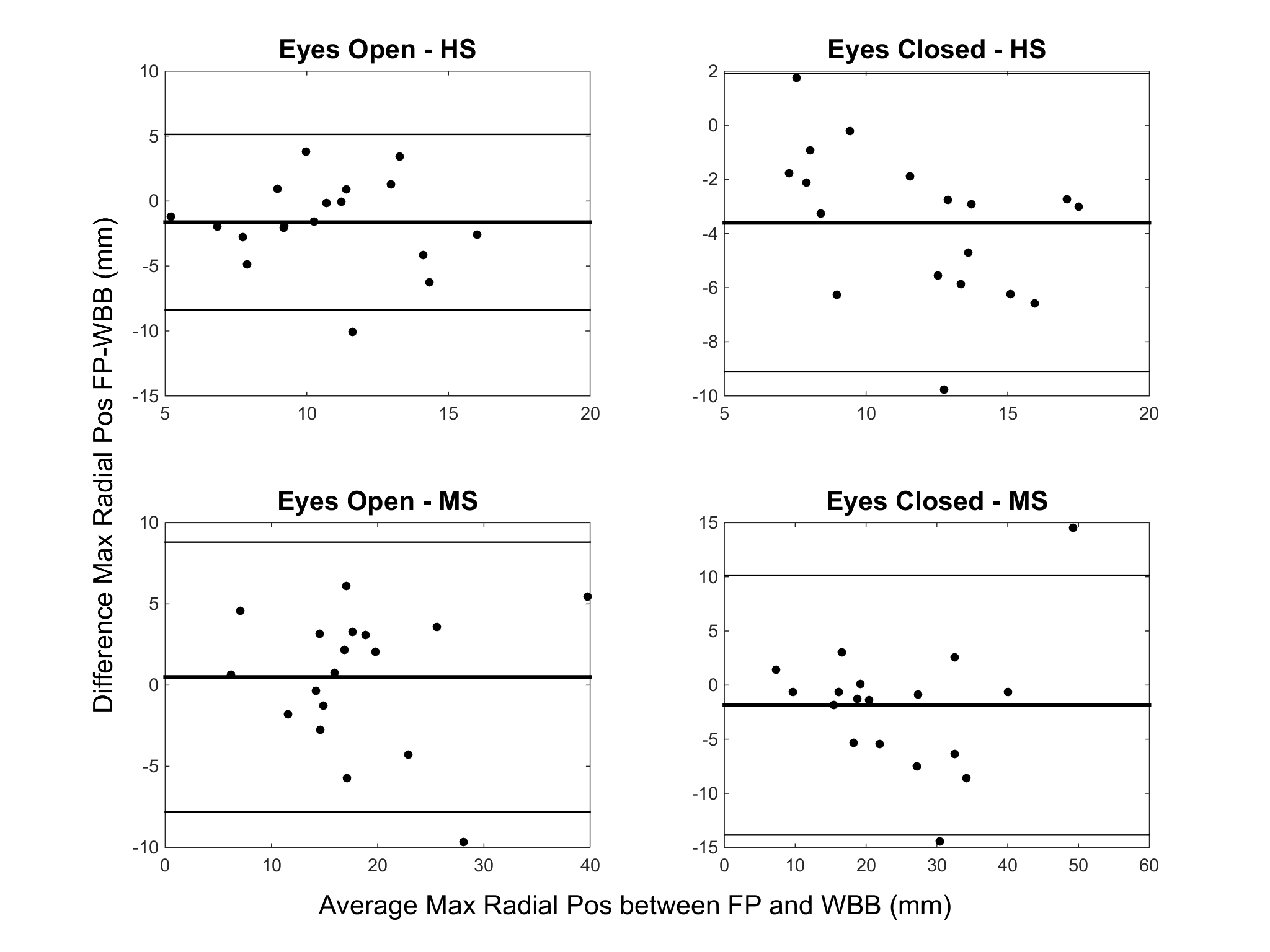

Supplement: Additional file 1: — S1-15. Bland-Altman Plots for all the features extracted from both the FP and the WBB. Y axis of each plot presents the difference between FP and WBB, while X axis presents the average between the two measures. The plots show a consistent trend of overestimation of the features extracted from the WBB data characterized by a negative bias (bold lane). Most features also present a linear trend whereas the difference between WBB and FP measurements increases with the magnitude of the feature. (ZIP 1255 kb) [file 12984_2017_230_MOESM1_ESM.zip › S11.png]

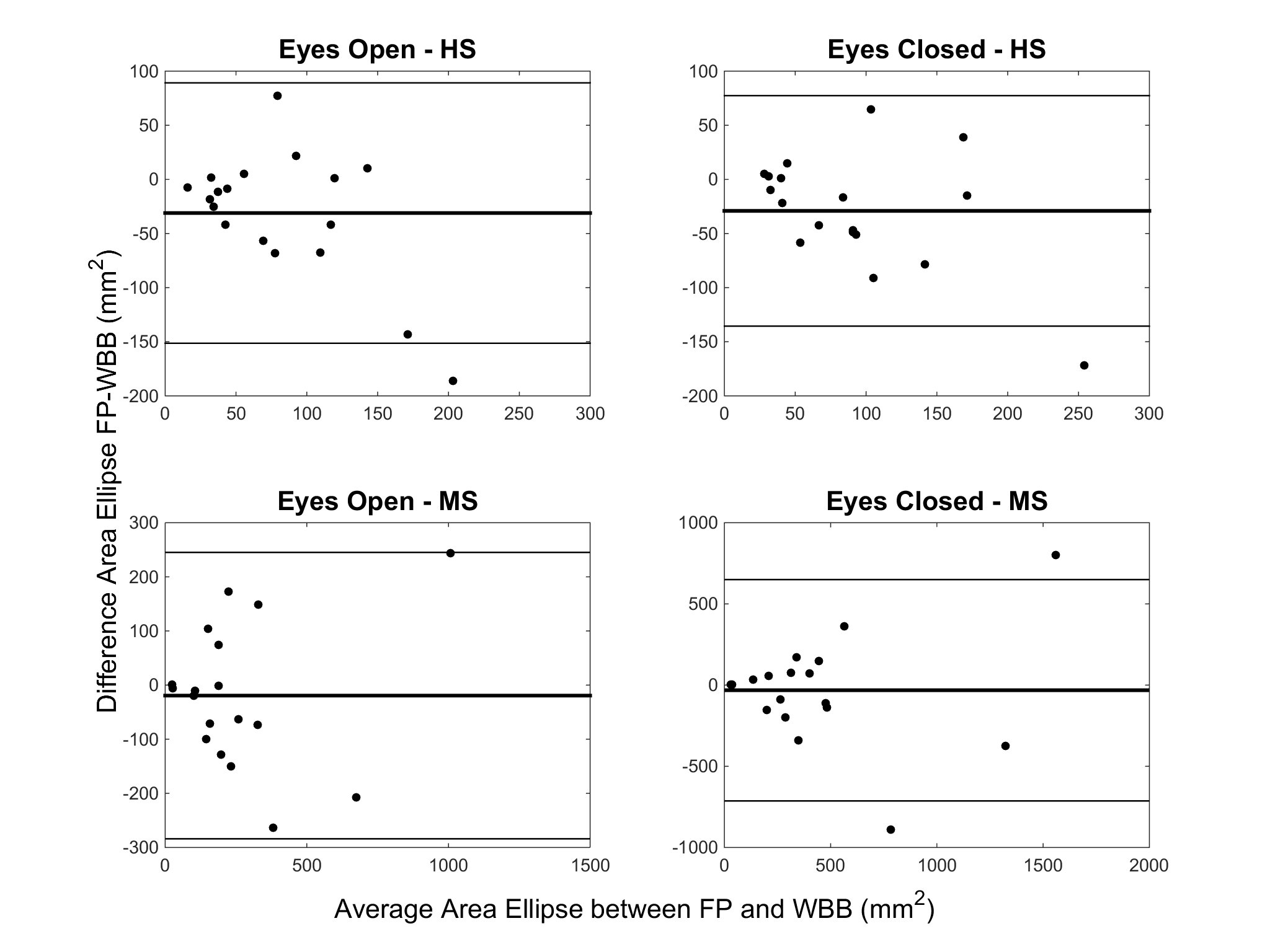

Supplement: Additional file 1: — S1-15. Bland-Altman Plots for all the features extracted from both the FP and the WBB. Y axis of each plot presents the difference between FP and WBB, while X axis presents the average between the two measures. The plots show a consistent trend of overestimation of the features extracted from the WBB data characterized by a negative bias (bold lane). Most features also present a linear trend whereas the difference between WBB and FP measurements increases with the magnitude of the feature. (ZIP 1255 kb) [file 12984_2017_230_MOESM1_ESM.zip › S12.png]

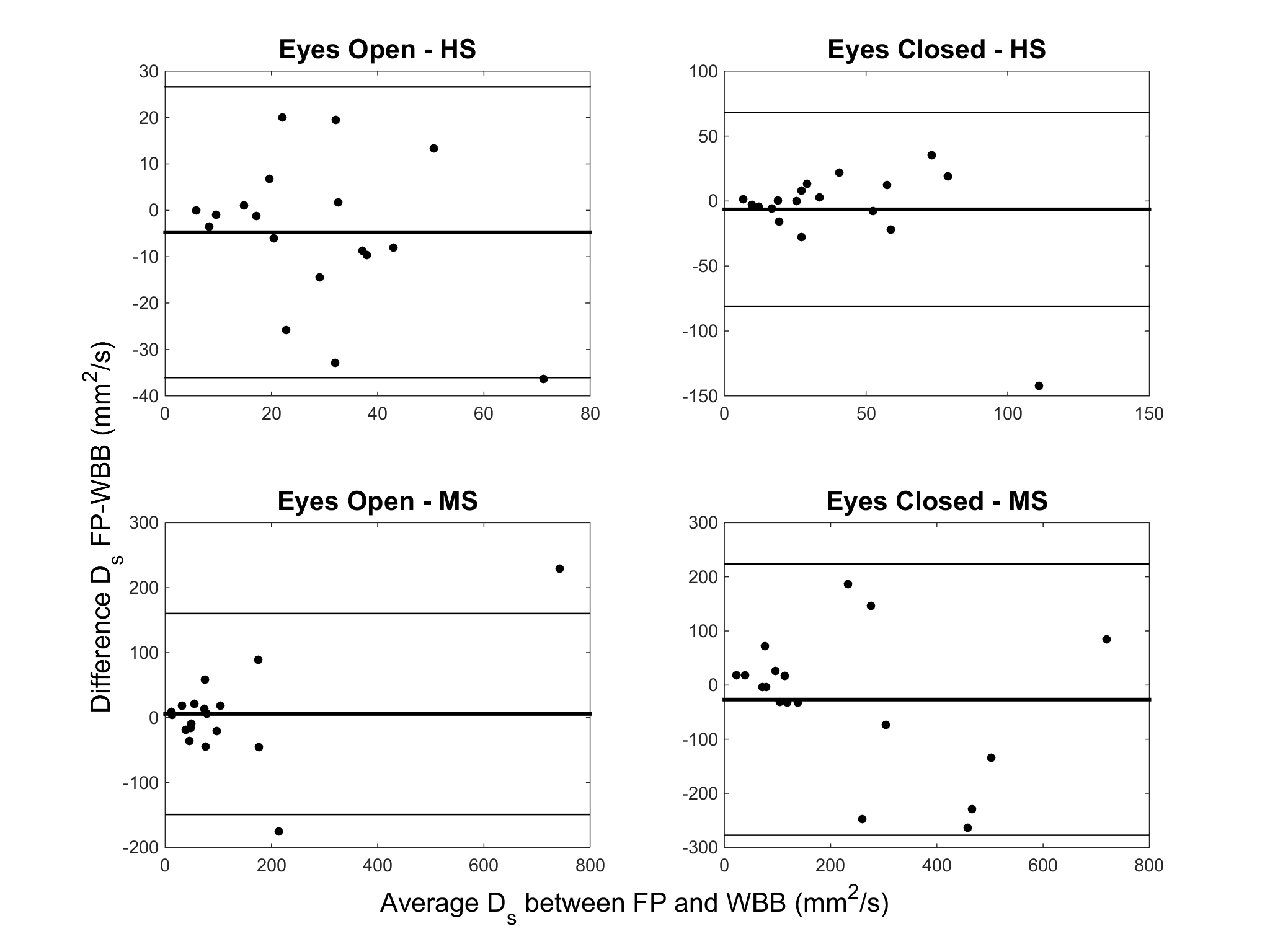

Supplement: Additional file 1: — S1-15. Bland-Altman Plots for all the features extracted from both the FP and the WBB. Y axis of each plot presents the difference between FP and WBB, while X axis presents the average between the two measures. The plots show a consistent trend of overestimation of the features extracted from the WBB data characterized by a negative bias (bold lane). Most features also present a linear trend whereas the difference between WBB and FP measurements increases with the magnitude of the feature. (ZIP 1255 kb) [file 12984_2017_230_MOESM1_ESM.zip › S13.png]

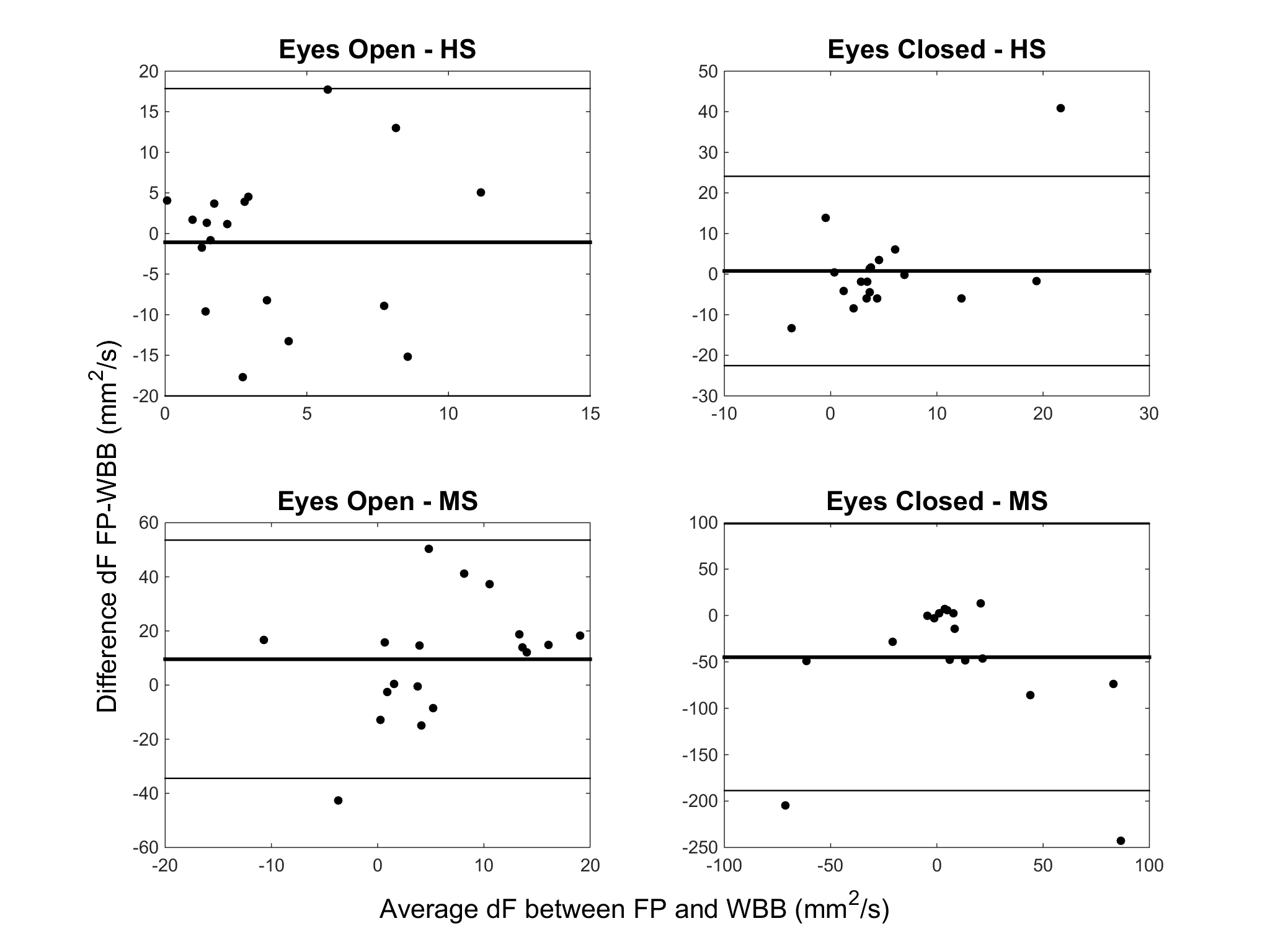

Supplement: Additional file 1: — S1-15. Bland-Altman Plots for all the features extracted from both the FP and the WBB. Y axis of each plot presents the difference between FP and WBB, while X axis presents the average between the two measures. The plots show a consistent trend of overestimation of the features extracted from the WBB data characterized by a negative bias (bold lane). Most features also present a linear trend whereas the difference between WBB and FP measurements increases with the magnitude of the feature. (ZIP 1255 kb) [file 12984_2017_230_MOESM1_ESM.zip › S14.png]

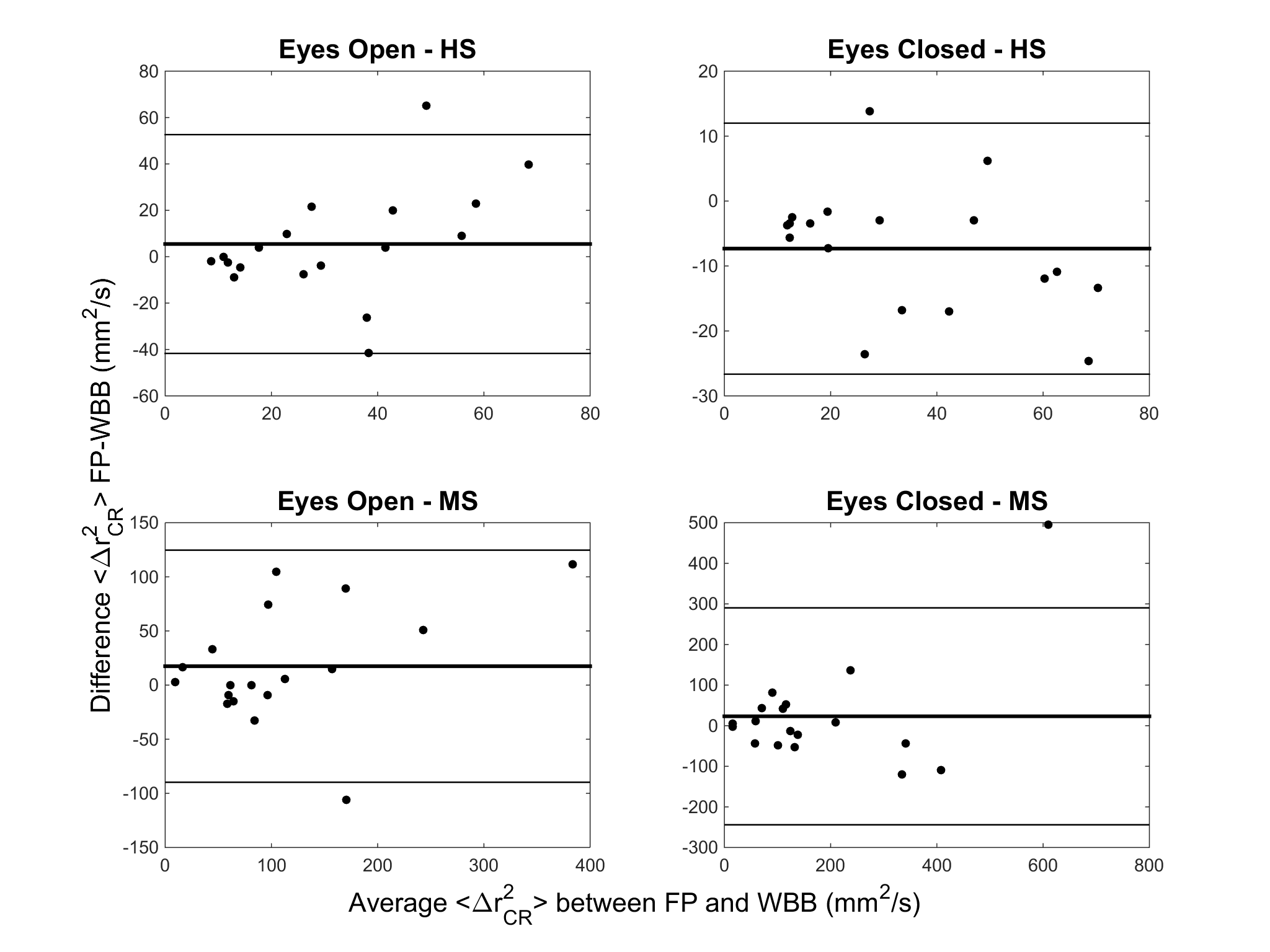

Supplement: Additional file 1: — S1-15. Bland-Altman Plots for all the features extracted from both the FP and the WBB. Y axis of each plot presents the difference between FP and WBB, while X axis presents the average between the two measures. The plots show a consistent trend of overestimation of the features extracted from the WBB data characterized by a negative bias (bold lane). Most features also present a linear trend whereas the difference between WBB and FP measurements increases with the magnitude of the feature. (ZIP 1255 kb) [file 12984_2017_230_MOESM1_ESM.zip › S15.png]

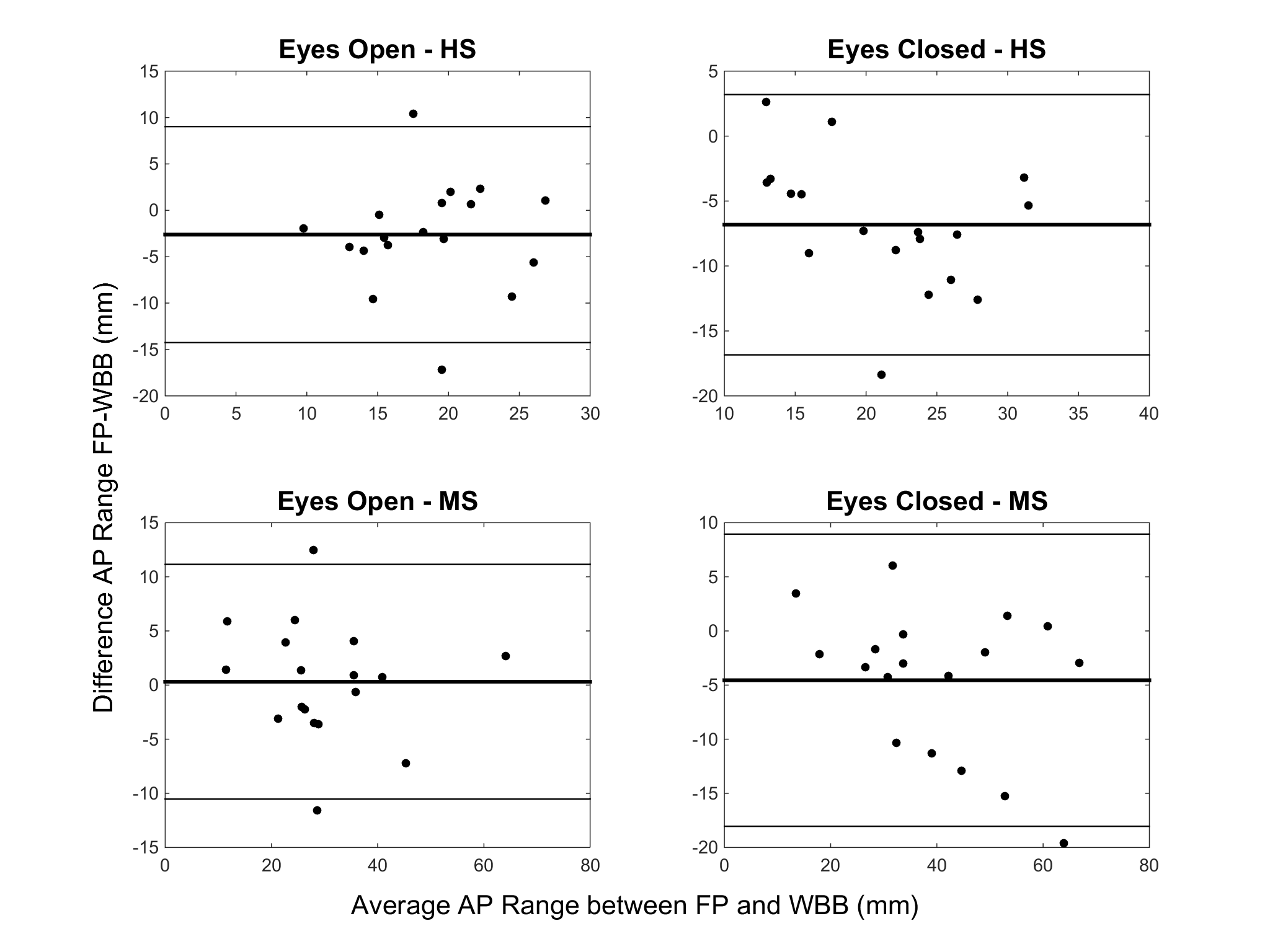

Supplement: Additional file 1: — S1-15. Bland-Altman Plots for all the features extracted from both the FP and the WBB. Y axis of each plot presents the difference between FP and WBB, while X axis presents the average between the two measures. The plots show a consistent trend of overestimation of the features extracted from the WBB data characterized by a negative bias (bold lane). Most features also present a linear trend whereas the difference between WBB and FP measurements increases with the magnitude of the feature. (ZIP 1255 kb) [file 12984_2017_230_MOESM1_ESM.zip › S2.png]

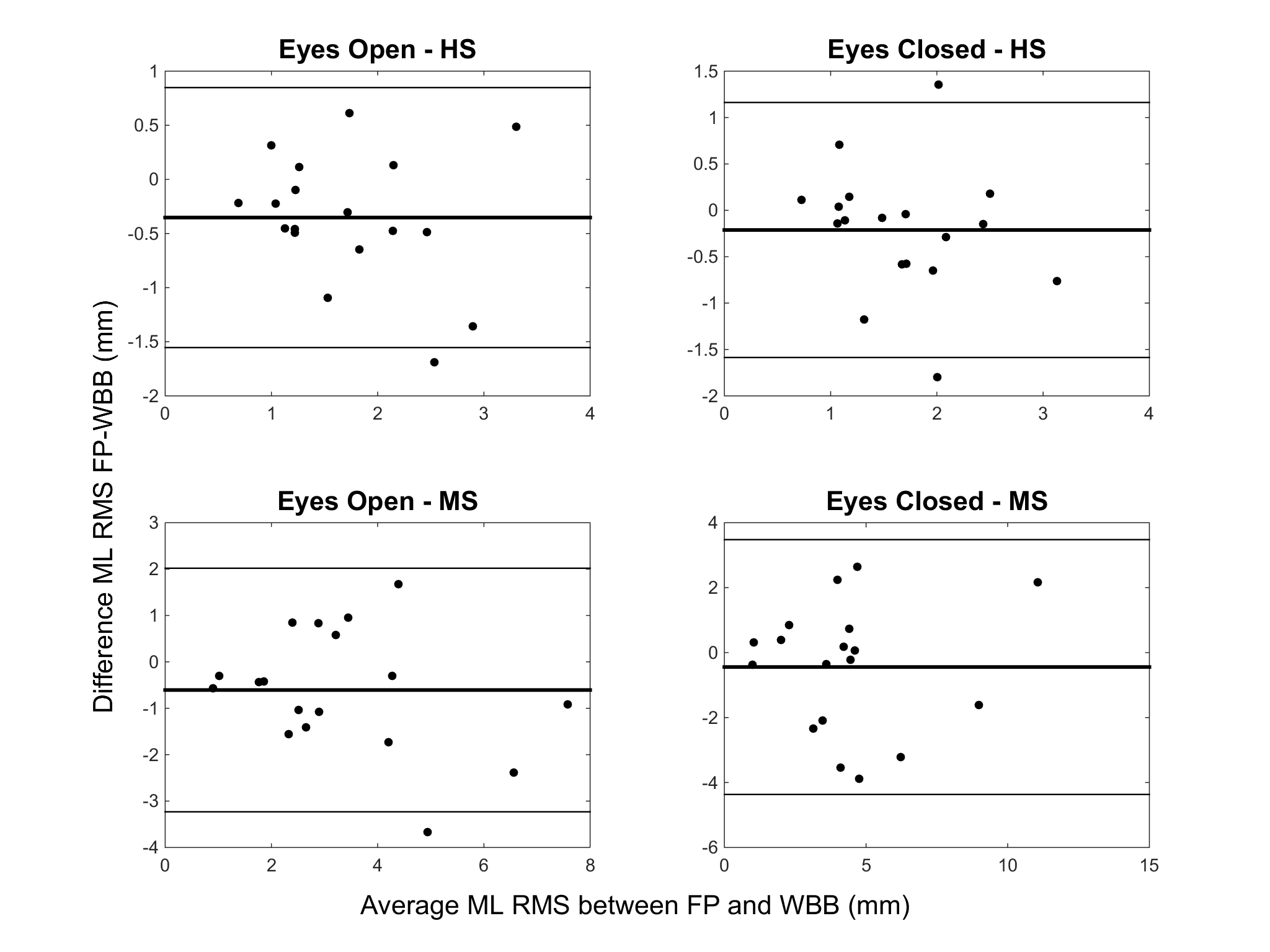

Supplement: Additional file 1: — S1-15. Bland-Altman Plots for all the features extracted from both the FP and the WBB. Y axis of each plot presents the difference between FP and WBB, while X axis presents the average between the two measures. The plots show a consistent trend of overestimation of the features extracted from the WBB data characterized by a negative bias (bold lane). Most features also present a linear trend whereas the difference between WBB and FP measurements increases with the magnitude of the feature. (ZIP 1255 kb) [file 12984_2017_230_MOESM1_ESM.zip › S3.png]

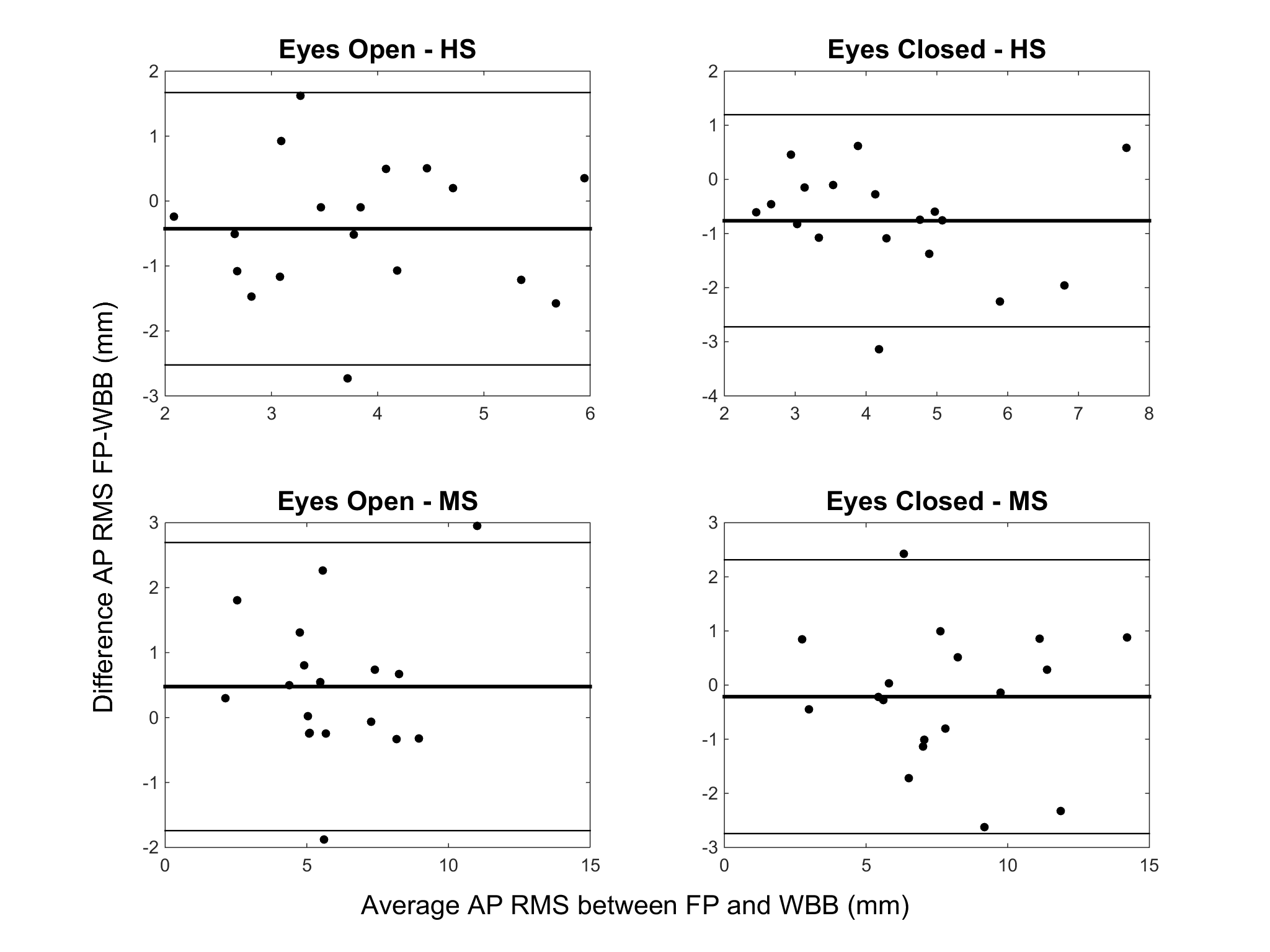

Supplement: Additional file 1: — S1-15. Bland-Altman Plots for all the features extracted from both the FP and the WBB. Y axis of each plot presents the difference between FP and WBB, while X axis presents the average between the two measures. The plots show a consistent trend of overestimation of the features extracted from the WBB data characterized by a negative bias (bold lane). Most features also present a linear trend whereas the difference between WBB and FP measurements increases with the magnitude of the feature. (ZIP 1255 kb) [file 12984_2017_230_MOESM1_ESM.zip › S4.png]

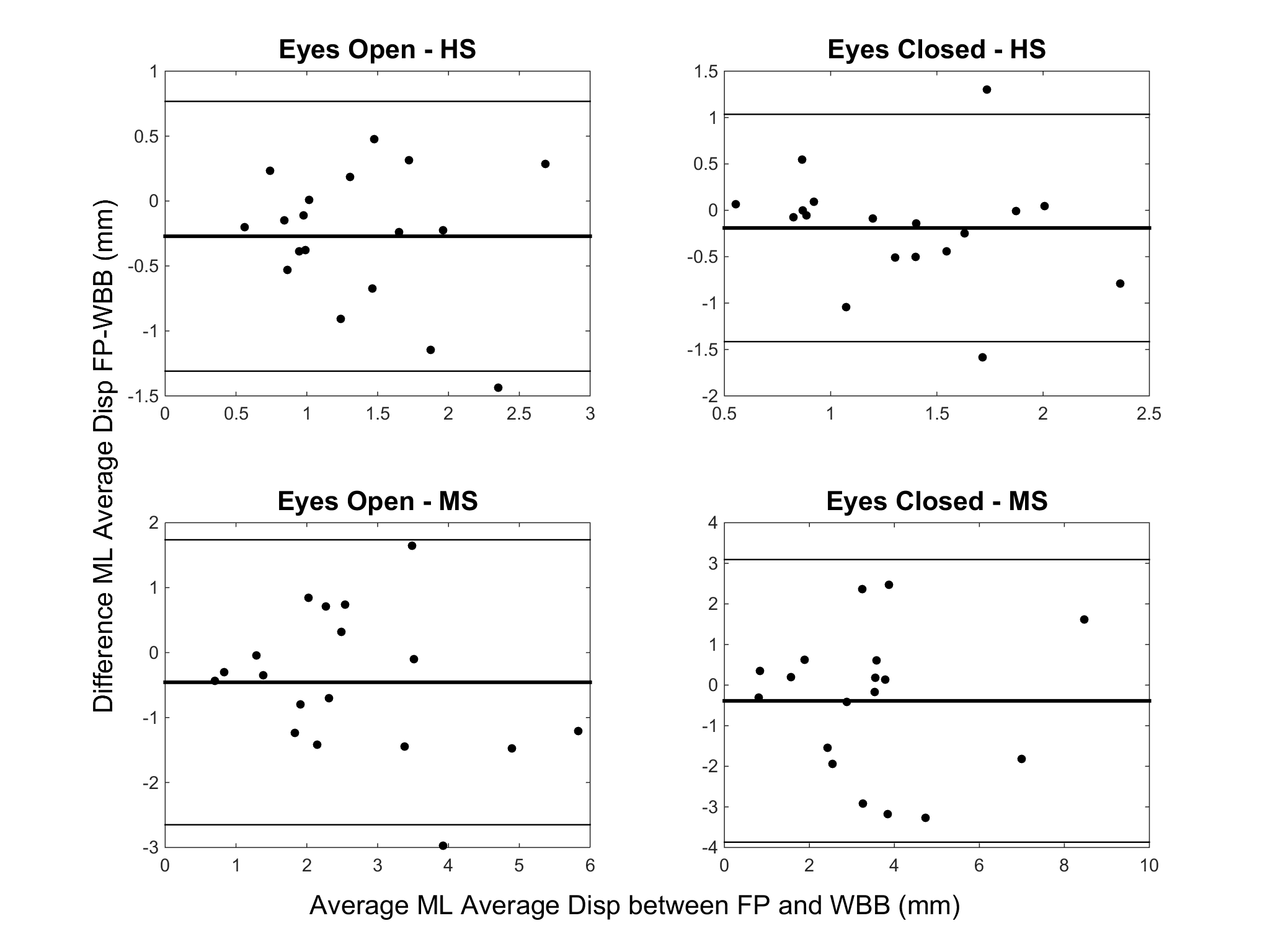

Supplement: Additional file 1: — S1-15. Bland-Altman Plots for all the features extracted from both the FP and the WBB. Y axis of each plot presents the difference between FP and WBB, while X axis presents the average between the two measures. The plots show a consistent trend of overestimation of the features extracted from the WBB data characterized by a negative bias (bold lane). Most features also present a linear trend whereas the difference between WBB and FP measurements increases with the magnitude of the feature. (ZIP 1255 kb) [file 12984_2017_230_MOESM1_ESM.zip › S5.png]

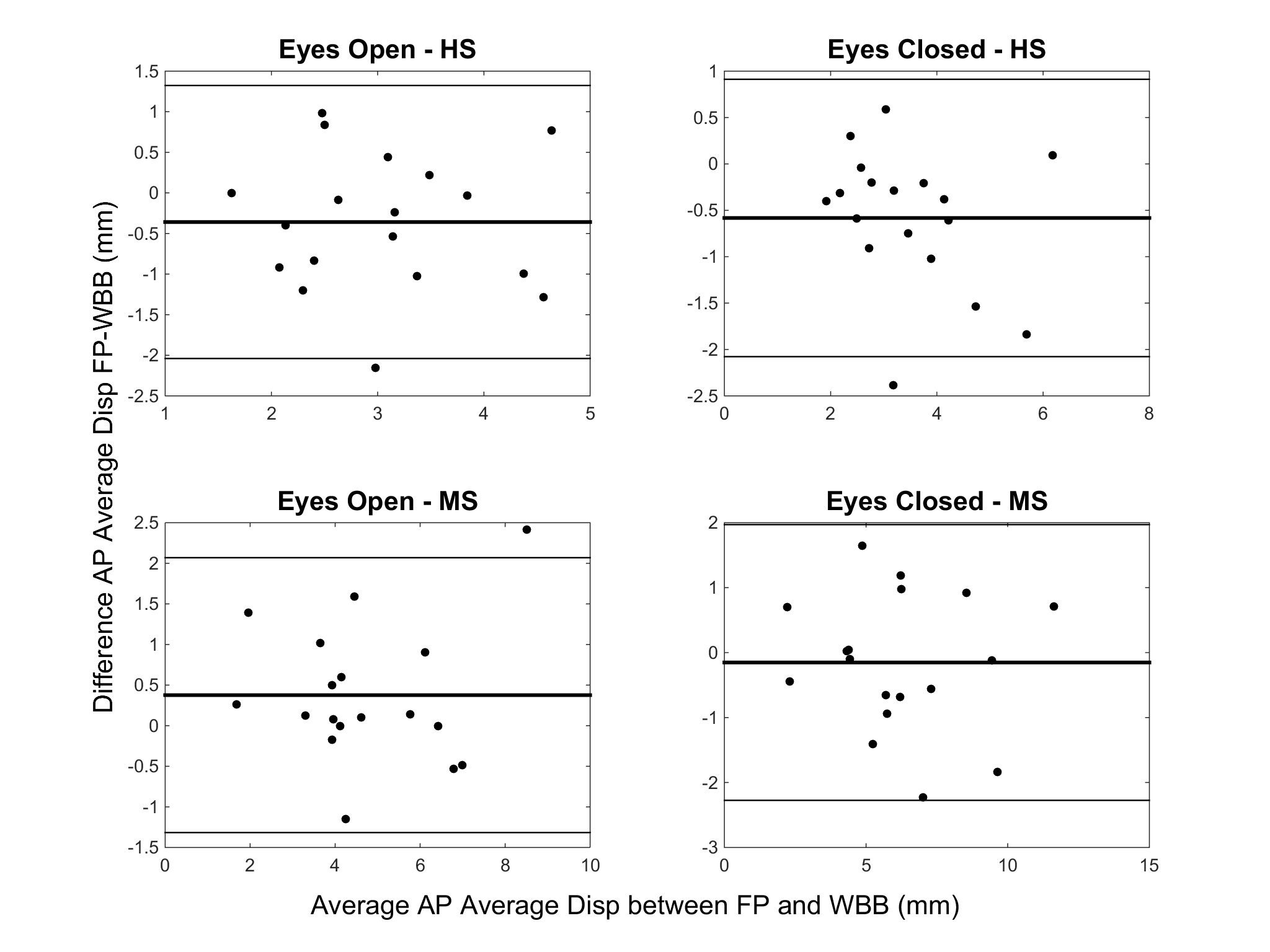

Supplement: Additional file 1: — S1-15. Bland-Altman Plots for all the features extracted from both the FP and the WBB. Y axis of each plot presents the difference between FP and WBB, while X axis presents the average between the two measures. The plots show a consistent trend of overestimation of the features extracted from the WBB data characterized by a negative bias (bold lane). Most features also present a linear trend whereas the difference between WBB and FP measurements increases with the magnitude of the feature. (ZIP 1255 kb) [file 12984_2017_230_MOESM1_ESM.zip › S6.png]

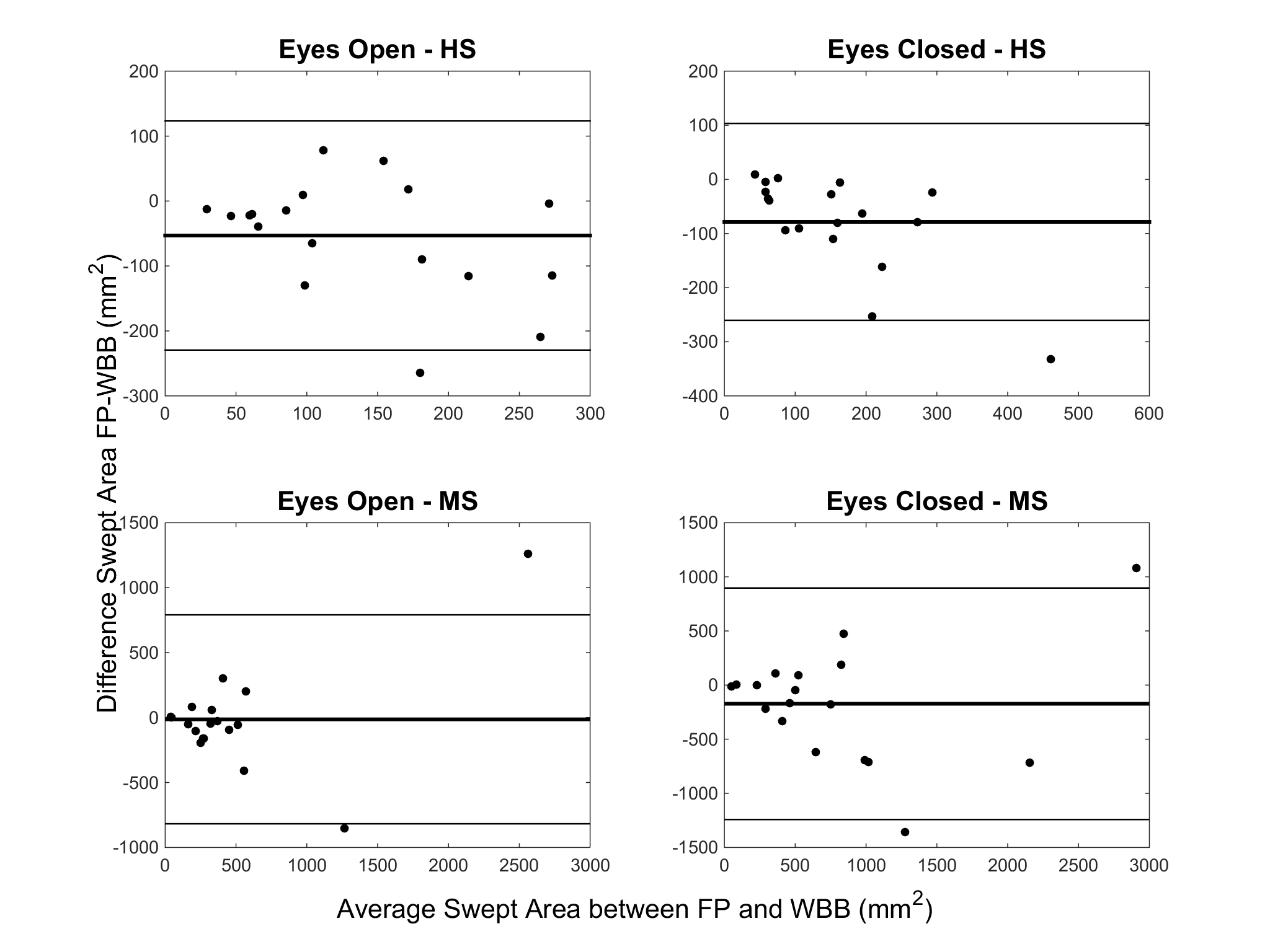

Supplement: Additional file 1: — S1-15. Bland-Altman Plots for all the features extracted from both the FP and the WBB. Y axis of each plot presents the difference between FP and WBB, while X axis presents the average between the two measures. The plots show a consistent trend of overestimation of the features extracted from the WBB data characterized by a negative bias (bold lane). Most features also present a linear trend whereas the difference between WBB and FP measurements increases with the magnitude of the feature. (ZIP 1255 kb) [file 12984_2017_230_MOESM1_ESM.zip › S7.png]

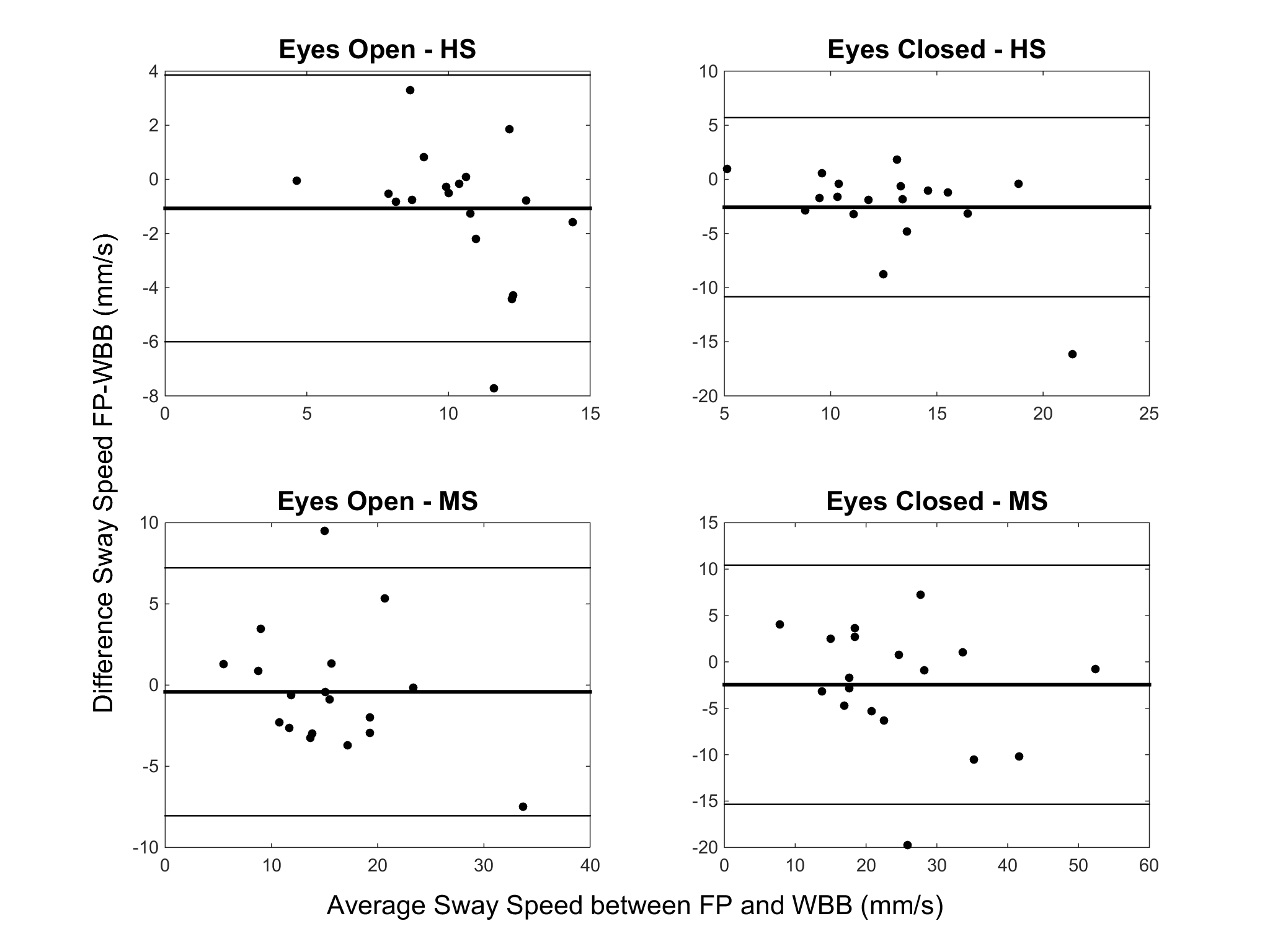

Supplement: Additional file 1: — S1-15. Bland-Altman Plots for all the features extracted from both the FP and the WBB. Y axis of each plot presents the difference between FP and WBB, while X axis presents the average between the two measures. The plots show a consistent trend of overestimation of the features extracted from the WBB data characterized by a negative bias (bold lane). Most features also present a linear trend whereas the difference between WBB and FP measurements increases with the magnitude of the feature. (ZIP 1255 kb) [file 12984_2017_230_MOESM1_ESM.zip › S8.png]

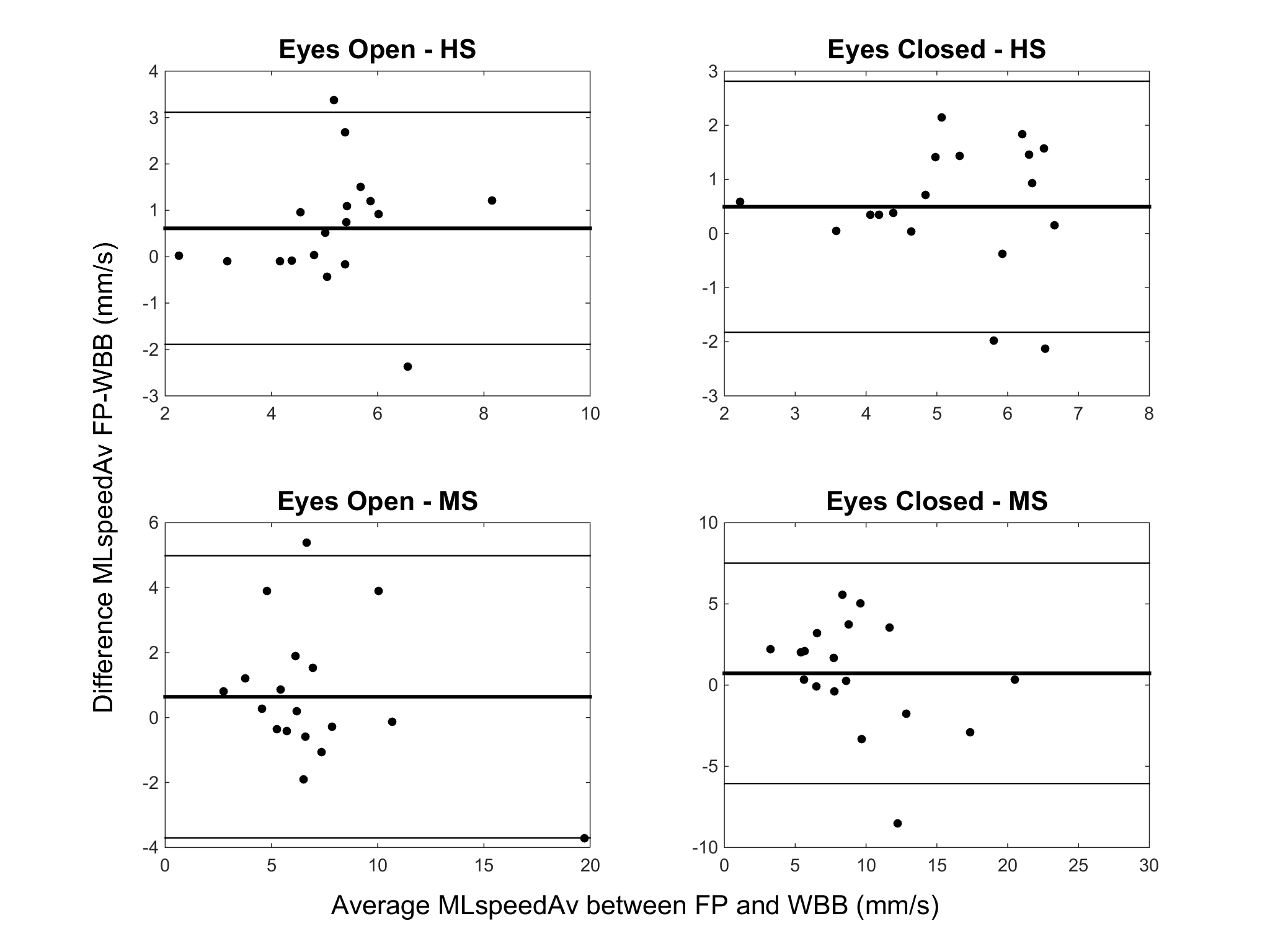

Supplement: Additional file 1: — S1-15. Bland-Altman Plots for all the features extracted from both the FP and the WBB. Y axis of each plot presents the difference between FP and WBB, while X axis presents the average between the two measures. The plots show a consistent trend of overestimation of the features extracted from the WBB data characterized by a negative bias (bold lane). Most features also present a linear trend whereas the difference between WBB and FP measurements increases with the magnitude of the feature. (ZIP 1255 kb) [file 12984_2017_230_MOESM1_ESM.zip › S9.png]
